# MeCP2 SUMOylation rescues *Mecp2*-mutant-induced behavioural deficits in a mouse model of Rett syndrome

**DOI:** 10.1038/ncomms10552

**Published:** 2016-02-04

**Authors:** Derek J. C. Tai, Yen C. Liu, Wei L. Hsu, Yun L. Ma, Sin J. Cheng, Shau Y. Liu, Eminy H. Y. Lee

**Affiliations:** 1 Institute of Biomedical Sciences, Academia Sinica, Taipei 115, Taiwan; 2 Graduate Institute of Life Sciences, National Defense Medical Center, Taipei 114, Taiwan; 3 Neuroscience Program in Academia Sinica, Taipei 115, Taiwan

## Abstract

The methyl-CpG-binding protein 2 (MeCP2) gene, *MECP2*, is an X-linked gene encoding the MeCP2 protein, and mutations of *MECP2* cause Rett syndrome (RTT). However, the molecular mechanism of *MECP2*-mutation-caused RTT is less known. Here we find that MeCP2 could be SUMO-modified by the E3 ligase PIAS1 at Lys-412. MeCP2 phosphorylation (at Ser-421 and Thr-308) facilitates MeCP2 SUMOylation, and MeCP2 SUMOylation is induced by NMDA, IGF-1 and CRF in the rat brain. MeCP2 SUMOylation releases CREB from the repressor complex and enhances *Bdnf* mRNA expression. Several *MECP2* mutations identified in RTT patients show decreased MeCP2 SUMOylation. Re-expression of wild-type MeCP2 or SUMO-modified MeCP2 in *Mecp2*-null neurons rescues the deficits of social interaction, fear memory and LTP observed in *Mecp2* conditional knockout (cKO) mice. These results together reveal an important role of MeCP2 SUMOylation in social interaction, memory and synaptic plasticity, and that abnormal MeCP2 SUMOylation is implicated in RTT.

## Abstract (web summary)

Post-translational modifications of methyl-CpG-binding protein 2 (MeCP2) are important for its function and dysfunction in Rett syndrome. Here, Tai *et al*. show a functional interaction between MeCP2 SUMOylation and phosphorylation in rodent behavior and synaptic plasticity.

## Graphical abstract

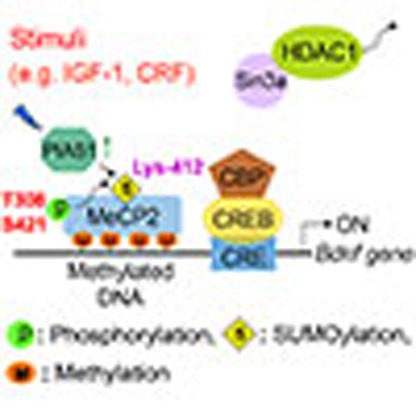

The methyl-CpG-binding protein 2 (MeCP2) gene, *MECP2*, is an X-linked gene encoding the MeCP2 protein that contains 486 amino acids with two major structurally conserved domains, the methyl-DNA-binding domain (MBD; 85 amino acids (a.a.) 78–162) and the transcriptional repression domain (104 amino acids, a.a. 207–310)^1^. In addition to MeCP2, there are four other proteins that also belong to the MBD family, including MBD1, MBD2, MBD3 and MBD4 (ref. 2), and they are highly conserved in the vertebrate. MeCP2 functions as a transcriptional repressor by binding to the CpG island of methylated DNA and by recruiting co-repressors, such as histone deacetylase 1 (HDAC1) and Sin3a (refs 3, 4). Later study indicates that MeCP2 is a multifunctional chromatin-associated protein that regulates the expression of various genes, and it could function as either a transcriptional repressor or activator^5^.

MeCP2 plays an important role in several neurodevelopmental disorders, such as Rett syndrome (RTT)^1^,^6^,^7^, which is an autism spectrum disorder caused by mutations of the *MECP2* gene and 218 mutations have been identified that are linked to RTT^8^. Among these mutations, there are three more common mutations, T158M, R168X (where X stands for a premature stop codon) and R133C, which account for ∼13, 12 and 7% of total RTT patients, respectively^9^. Twofold decrease in methyl-DNA binding was found in T158M mutation compared with wild-type MeCP2, and T158M mutation is the most common and severe mutation in RTT patients^10^. R168X is a truncated mutant MeCP2 protein that lacks the C-terminal domain; thus, it cannot interact with other proteins and oligomer chromatin cannot be formed on target genes^11^. For R133C, its methyl-DNA binding was totally absent; however, it still retains the ability to interact with other proteins and chromatin DNA^11^. Patients with RTT usually develop normally before 18 months of age, but abnormal behaviours and regression develop afterwards that often include motor and language deficits, cognitive impairment, mental retardation and autism-like behaviours^1^,^12^,^13^. Similar behavioural impairments were seen in mice with truncated MeCP2 (refs 14, 15) and in mouse model of RTT with MeCP2 mutations at T158 and R306 (refs 16, 17). Moreover, learning and memory function as well as synaptic plasticity were found impaired in a truncated MeCP2 mouse model of RTT^18^.

Protein phosphorylation is well studied with MeCP2. The first identified phosphorylation site on MeCP2 is Ser-421. MeCP2 phosphorylation at Ser-421 is induced by neuronal activation in the brain through CaMKII-dependent signalling, and it is involved in dendritic growth, spine maturation and brain-derived neurotrophic factor (BDNF) gene expression^19^. Phosphorylation of Ser-80 of MeCP2 was identified in epileptic brains from human, rat and mouse, and MeCP2 phosphorylation at this residue maintains its chromatin association with the gene promoter for transcriptional regulation^20^. More recently, MeCP2 was also found to be phosphorylated at Thr-308 by neuronal activation, and MeCP2 phosphorylation at Thr-308 disrupts its interaction with the nuclear receptor co-repressor complex and abolishes the repression activity of MeCP2 (ref. 21).

Post-translational modification of proteins with small ubiquitin-like modifier (SUMO) is an important mechanism in the regulation of various cellular functions^22^,^23^. We further showed that protein SUMOylation is important for long-term memory formation^24^,^25^. Post-translational modifications with MeCP2 were also reported^26^. MeCP2 was found to be SUMO-modified at Lys-223, and MeCP2 SUMOylation at this residue is necessary for its transcriptional repression activity and synapse development^27^. There are many lysine residues on MeCP2 and one consensus SUMO–substrate motif (ψ-K-X-E, where ψ stands for a hydrophobic amino acid) was identified (Lys-363), which implicate that MeCP2 may be sumoylated at other lysine residues also. In this study, we aimed to identify the candidate SUMO sites on MeCP2 and examine the molecular mechanism of MeCP2 SUMOylation and its relationship with RTT. Our results show that MeCP2 SUMOylation rescues the behavioural and synaptic deficits in *Mecp2* conditional knockout (cKO) mice.

## Results

### Identification of candidate SUMO sites on MeCP2

**Figure 1:**
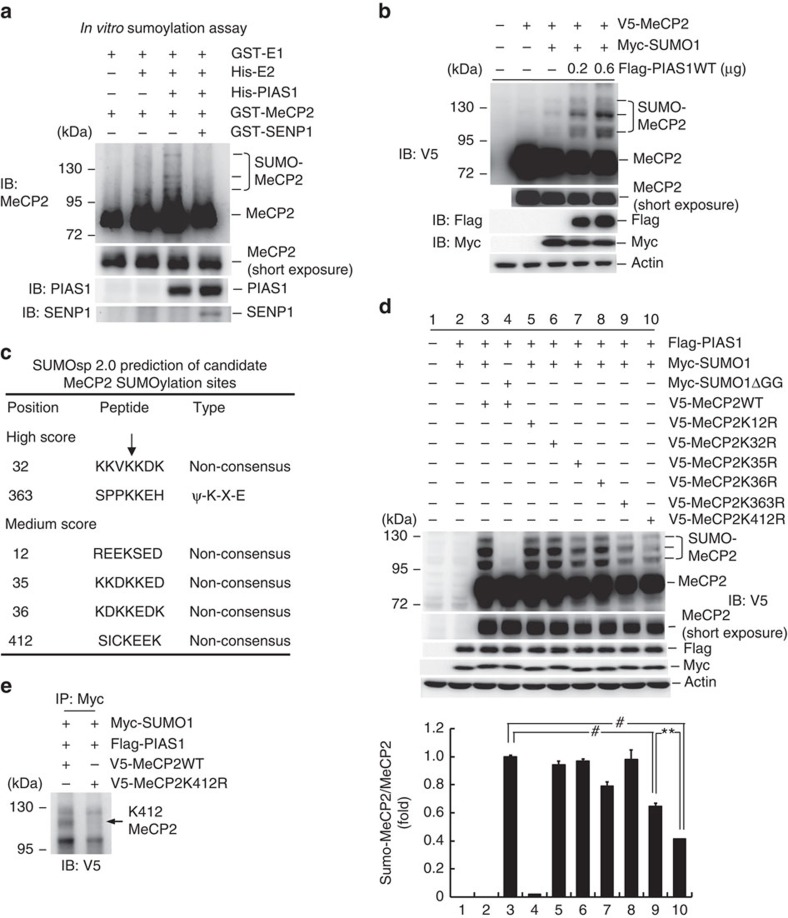
Identification of candidate SUMO sites on MeCP2. (**a**) *In vitro* SUMOylation assay showing MeCP2 SUMOylation by PIAS1. Purified GST-E1, His-E2, His-PIAS1, GST-MeCP2 and GST-SENP1 proteins were added to the reaction for this assay. (**b**) V5-MeCP2 and Myc-SUMO1 plasmids were co-transfected with different amounts of Flag-PIAS1 plasmid to HEK293T cells confirming MeCP2 SUMOylation by PIAS1. (**c**) SUMO2.0 Software prediction of candidate SUMO acceptors on MeCP2. The ‘K' letter indicated by the arrow represents the candidate SUMO sites. (**d**) Flag-PIAS1 and Myc-SUMO1 (or Myc-SUMO1ΔGG) plasmids were co-transfected with V5-MeCP2WT or different V5-MeCP2 lysine mutant plasmids to HEK293T cells. MeCP2 SUMOylation was examined by immunoblotting using anti-V5 antibody. The quantified result is shown in the lower panel (*n*=2 each group; F_9,10_=240.59, #*P*<0.001; *q*=12.73, #*P*<0.001 comparing lane 3 versus lane 9; *q*=21.1, #*P*<0.001 comparing lane 3 versus lane 10; *q*=8.37, **P<0.01 comparing lane 9 versus lane 10, one-way ANOVA followed by Newman–Keul *post hoc* multiple comparisons). (**e**) V5-MeCP2 or V5-MeCP2K412R plasmid was co-transfected with Myc-SUMO1 and Flag-PIAS1 plasmids to HEK293T cells. The cell lysate was immunoprecipitated with anit-Myc antibody and immunoblotted with anti-V5 antibody for confirmation of MeCP2 SUMOylation at Lys-412. The arrow indicates MeCP2 SUMOylation at Lys-412. All experiments are in two repeats. Data are expressed as mean±s.e.m.

To examine whether MeCP2 could be SUMO-modified, we first performed *in vitro* SUMOylation assay. Recombinant E1, E2, protein inhibitor of activated STAT1 (PIAS1) and MeCP2 proteins (His- or glutathione *S*-transferase (GST)-tagged) were added to the reaction and western blot analysis against MeCP2 and PIAS1 was carried out. Results revealed that MeCP2 SUMOylation was observed when E1, E2, PIAS1 and MeCP2 proteins were added; however, this effect was blocked by the addition of sentrin-specific protease 1 (SENP1), an enzyme that removes the sumo molecule from sumo-conjugated protein (Fig. 1a). Next, we examined whether MeCP2 could be sumoylated by PIAS1 in the cell. V5-MeCP2, Myc-SUMO1 and Flag-PIAS1 (in different amount) plasmids were transfected to HEK293T cells and *in vitro* SUMOylation assay was carried out. Results revealed that PIAS1 enhanced the SUMOylation of MeCP2 in a dose-dependent manner (Fig. 1b). Next, we determined the candidate SUMO acceptors on MeCP2, and mass spectrometry (MS) was carried out. The MS result revealed 10 candidate SUMO residues on MeCP2; however, none of them fits to the consensus SUMO–substrate motif. We then adopted the bioinformatics method and SUMO2.0 Software for further analysis. Results revealed two lysine residues that show high score, and one of them (Lys-363) fits to the consensus SUMO–substrate motif. Four additional lysine residues show medium score (Fig. 1c). We have generated individual mutants against these six residues and transfected each mutant (V5-tagged), together with Flag-PIAS1 and Myc-SUMO1, to HEK293T cells for further examination. Results revealed that MeCP2 SUMOylation was observed when V5-MeCP2WT was transfected; however, this effect was blocked when Myc-SUMO1ΔGG, the SUMO1 plasmid that lacks the C-terminal di-glycine motif essential for SUMO1 conjugation^28^, was transfected (Fig. 1d). Transfection of the V5-MeCP2K12R, V5-MeCP2K32R, V5-MeCP2K35R and V5-MeCP2K36R did not alter MeCP2 SUMOylation. Transfection of V5-MeCP2K363R diminished MeCP2 SUMOylation and blocked the upper MeCP2 sumo-band (Fig. 1d); however, transfection of V5-MeCP2K412R prevented both the upper and middle MeCP2 sumo-bands (Fig. 1d). The overall SUMOylation intensity was significantly reduced by V5-MeCP2K412R transfection than by V5-MeCP2K363R transfection (Fig. 1d). Next, we confirmed MeCP2 SUMOylation at Lys-412 in the cell. Flag-PIAS1, Myc-SUMO1, V5-MeCP2WT or V5-MeCP2K412R plasmids were transfected to HEK293T cells. The cells were immunoprecipitated with anti-Myc antibody and immunoblotted with anti-V5 antibody. Result revealed that the middle sumo-band indicates the Lys-412 residue, and the SUMOylation intensity was decreased by V5-MeCP2K412R transfection (Fig. 1e). Because MeCP2K412R showed more significant effect in decreasing MeCP2 SUMOylation than MeCP2K363R did, we have focused on Lys-412 in the present study. Besides, an earlier report showed that MeCP2 could be SUMO-modified at Lys-223 (ref. 27); we also examined MeCP2 SUMOylation at this residue. V5-MeCP2WT or V5-MeCP2K223R was co-transfected with Flag-PIAS1 and Myc-SUMO1 to HEK293T cells, and MeCP2 SUMOylation was examined. V5-MeCP2K412R was transfected as a positive control. Result revealed that MeCP2 SUMOylation was not altered by V5-MeCP2K223R transfection (Supplementary Fig. 2a).

### MeCP2 can be SUMO-modified by PIAS1 in the hippocampus

**Figure 2:**
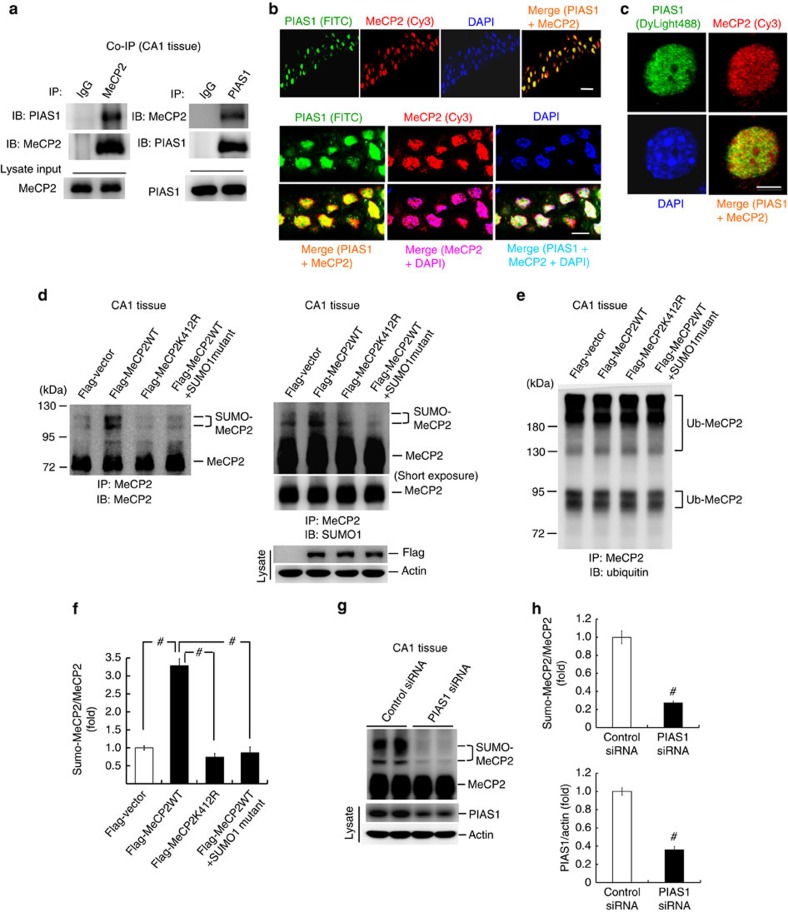
MeCP2 is associated with PIAS1 and can be sumoylated by PIAS1 at Lys-412 in rat hippocampus. (**a**) Co-IP experiment showing PIAS1 association with MeCP2 and *vice versa* in rat CA1 area. Experiments are in two repeats. (**b**) Immunohistochemistry showing PIAS1 and MeCP2 are both present in the nucleus of the same neurons in the CA1 area of the rat brain. *N*=3. Scale bar, 25 μm for the upper panel and scale bar, 10 μm for the lower panel. (**c**) Immunocytochemistry showing PIAS1 and MeCP2 are colocalized in the nucleus of the same neuron from rat hippocampal culture. Scale bar, 5 μm. Experiments are in two repeats. (**d**) Flag-vector, Flag-MeCP2WT (with or without the addition of SUMO1-mutant protein) and Flag-MeCP2K412R plasmids were transfected to rat CA1 area, and *in vitro* SUMOylation assay was carried out 48 h later to determine MeCP2 SUMOylation at Lys-412 in the hippocampus. Left panel: immunoblotted with anti-MeCP2 antibody. Right panel: immunoblotted with anti-SUMO1 antibody. Plasmid transfection and expression was confirmed with western blot analysis using anti-Flag antibody. The quantified result is shown in **f** (*n*=4 each group; F_3,12_=76.75, #*P*<0.001; *q*=16.5, #*P*<0.001 comparing the Flag-MeCP2WT group with Flag-vector group; *q*=18.39, #*P*<0.001 comparing the Flag-MeCP2K412R group with Flag-MeCP2WT group; *q*=17.47, #*P*<0.001 comparing the Flag-MeCP2WT+SUMO1-mutant group with Flag-MeCP2WT group, one-way ANOVA followed by *post hoc* Newman–Keuls multiple comparisons). (**e**) Cell lysates from the same plasmid transfections as described in **d** were immunoprecipitated with anti-MeCP2 antibody and immunoblotted with anti-ubiquitin antibody. Ub-MeCP2: ubiquitinated MeCP2. (**g**) Control siRNA or PIAS1 siRNA (8 pmol) was transfected to the CA1 area in the rat brain and endogenous MeCP2 SUMOylation was determined using *in vitro* SUMOylation assay. (**h**) The quantified result of MeCP2 SUMOylation is shown in the upper panel (*n*=6 each group; *t*_1,10_=9.65, #*P*<0.001, Student's *t*-test). The level of PIAS1 expression after PIAS1 siRNA transfection was determined by western blot analysis, and the quantified result is shown in the lower panel (*n*=6 each group; *t*_1,10_=11.38, #*P*<0.001, Student's *t*-test). Data are expressed as mean±s.e.m.

The above results showed that MeCP2 could be sumoylated by PIAS1 at Lys-412 in the cell; here we examined whether MeCP2 could be SUMO-modified by PIAS1 in the hippocampus of the rat brain. Co-immunoprecipitation (co-IP) was first carried out. Results revealed that when cells were immunoprecipitated with anti-MeCP2 antibody and immunoblotted with anti-PIAS1 antibody, MeCP2 is apparently associated with PIAS1 (Fig. 2a, left panel). Similar results were obtained when cells were immunoprecipitated with anti-PIAS1 antibody and immunoblotted with anti-MeCP2 antibody (Fig. 2a, right panel). Next, we examined whether PIAS1 and MeCP2 are present in the same hippocampal neurons. Brain sections containing the CA1 region were subjected to immunohistochemistry staining. Results revealed that the immunofluorescence for PIAS1 (green), MeCP2 (red) and 4,6-diamidino-2-phenylindole (DAPI; blue) was visualized in CA1 neurons (Fig. 2b, upper panel). When CA1 neurons were visualized at a higher magnification, PIAS1 and MeCP2 were found both to be present and colocalized in the nucleus of the same hippocampal neurons (Fig. 2b, lower panel). For better visualization of PIAS1 and MeCP2 distribution in individual neurons, cultured hippocampal neurons were subjected to immunocytochemistry staining of PIAS1 and MeCP2. Results revealed that PIAS1 and MeCP2 are both present and colocalized in the nucleus of the same hippocampal neuron, and puncta distribution of MeCP2 was observed at heterochromatins (Fig. 2c). Next, we examined whether MeCP2 could be sumoylated at Lys-412 in the hippocampus. Flag-vector, Flag-MeCP2WT or Flag-MeCP2K412R was transfected to the rat CA1 area and *in vitro* SUMOylation assay was performed 48 h later. Results indicated that transfection of Flag-MeCP2WT significantly increased the level of MeCP2 SUMOylation, but this effect was blocked by Flag-MeCP2K412R transfection. Addition of the SUMO1-mutant protein also blocked the enhancing effect of Flag-MeCP2WT on MeCP2 SUMOylation (compared with the Flag-MeCP2WT group; Fig. 2d, left panel). These results were confirmed when cells were immunoprecipitated with anti-MeCP2 antibody and immunoblotted with anti-SUMO1 antibody (Fig. 2d, right panel). Plasmid transfection and expression was confirmed with western blot analysis using anti-Flag antibody (Fig. 2d, lower-right panel). Because both SUMOylation and ubiquitination occur at lysine residues, we further examined whether the observed SUMO–MeCP2 bands could be ubiquitinated MeCP2. The same cell lysates were immunoprecipitated with anti-MeCP2 antibody but immunoblotted with anti-ubiquitin antibody. Result revealed that the ubiquitinated MeCP2 bands showed molecular weight below 95 kDa or above 130 kDa (Fig. 2e), different from that of the sumoylated MeCP2 bands, which are located between 95 and 130 kDa (Fig. 2d). The quantified result of MeCP2 SUMOylation is shown in Fig. 2f. Plasmid transfection and expression in CA1 neurons was further confirmed by immunohistochemistry using anti-Flag antibody and fluorescein isothiocyanate (FITC)-conjugated secondary antibody (Supplementary Fig. 3a,b).

We next addressed the issue whether MeCP2 is sumoylated by PIAS1 endogenously. Rats received control short interfering RNA (siRNA) or PIAS1 siRNA (8 pmol) transfection to their CA1 area, and endogenous MeCP2 SUMOyation was determined using *in vitro* SUMOylation assay 48 h later. Results revealed that knockdown of PIAS1 significantly decreased the level of endogenous MeCP2 SUMOylation (Fig. 2g). The quantified result is shown in Fig. 2h (upper panel). The effectiveness of PIAS1 siRNA transfection was confirmed by decreased PIAS1 expression in the CA1 area (Fig. 2h, lower panel).

### MeCP2 phosphorylation facilitates MeCP2 SUMOylation

**Figure 3:**
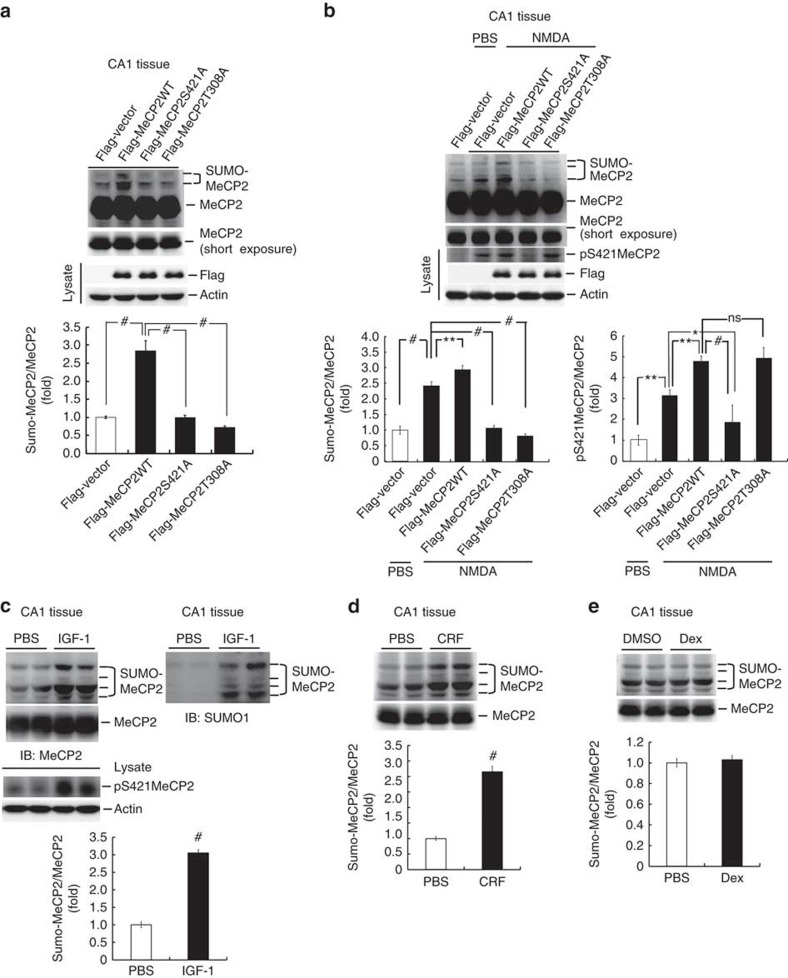
MeCP2 phosphorylation facilitates MeCP2 SUMOylation and MeCP2 SUMOylation is induced by NMDA and IGF-1 and by CRF treatments in the hippocampus. (**a**) Flag-vector, Flag-MeCP2WT, Flag-MeCP2S421A or Flag-MeCP2T308A plasmid was transfected to rat CA1 area, and MeCP2 SUMOylation was determined by *in vitro* SUMOylation assay and quantified (*n*=4 each group; F_3,12_=40.85, #*P*<0.001; *q*=12.02, #*P*<0.001, Flag-MeCP2WT group versus Flag-vector group; *q*=12.1, #*P*<0.001, Flag-MeCP2S421A group versus Flag-MeCP2WT group; *q*=13.88, #*P*<0.001, Flag-MeCP2T308A group versus Flag-MeCP2WT group). (**b**) The same plasmids were transfected to rat CA1 area as described above. NMDA (2 μg μl^−1^) was administered 47 h after plasmid transfection. An additional group with Flag-vector transfection+PBS injection served as the control group. MeCP2 SUMOylation was determined by *in vitro* SUMOylation assay, and MeCP2 phosphorylation at Ser-421 was determined by western blot analysis using anti-phospho-Ser421MeCP2 antibody 1 h after NMDA injection (for MeCP2 SUMOylation, *n*=4 each group; F_4,15_=74.65, #*P*<0.001; *q*=12.7, #*P*<0.001, Flag-vector+NMDA group versus Flag-vector+PBS group; *q*=4.65, ^**^P<0.01, Flag-MeCP2WT+NMDA group versus Flag-vector+NMDA group; *q*=12.15, #*P*<0.001, Flag-MeCP2S421A+NMDA group versus Flag-vector+NMDA group; *q*=14.42, #*P*<0.001, Flag-MeCP2T308A+NMDA group versus Flag-vector+NMDA group. For MeCP2 phosphorylation at Ser-421, *n*=4 each group; F_4,15_=23.65, #*P*<0.001; *q*=5.97, ^**^P<0.01 comparing the Flag-vector+NMDA group with Flag-vector+PBS group; *q*=4.59, ^**^P<0.01, Flag-MeCP2WT+NMDA group versus Flag-vector+NMDA group; *q*=8.17, #*P*<0.001, Flag-MeCP2S421A+NMDA group versus Flag-MeCP2WT+NMDA group; *q*=3.58, *P<0.05, Flag-MeCP2S421A+NMDA group versus Flag-vector+NMDA group). ns: non-significant. (**c**) PBS or IGF-1 (100 ng ml^−1^) was injected to rat CA1 area, and MeCP2 SUMOylation was determined 1 h later. Left panel: immunoblotted with anti-MeCP2 antibody. Right panel: immunoblotted with anti-SUMO1 antibody (*n*=6 each group; *t*_1,10_=15.9, #*P*<0.001). (**d**) PBS or CRF (100 ng μl^−1^) was injected to rat CA1 area, and MeCP2 SUMOylation was determined 1 h later (*n*=6 each group; *t*_1,10_=10.91, #*P*<0.001). (**e**) DMSO or dexamethasone (Dex, 30 ng μl^−1^) was injected to rat CA1 area and MeCP2 SUMOylation was determined 1 h later (*n*=6 each group; *t*_1,10_=0.57, *P*>0.05). One-way ANOVA followed by *post hoc* Newman–Keuls multiple comparisons (**a**,**b**) or Student's *t*-test (**c**–**e**). Data are expressed as mean±s.e.m.

The above results demonstrated that MeCP2 could be sumoylated by PIAS1 in the hippocampus; here we examined the mechanism of MeCP2 SUMOylation. Previous studies have shown that MeCP2 phosphorylation at Ser-421 and Thr-308 is induced by neuronal activation^19^,^21^. Here we examined whether MeCP2 phosphorylation may facilitate MeCP2 SUMOylation. Rats received Flag-vector, Flag-MeCP2WT, Flag-MeCP2S421A or Flag-MeCP2T308A transfection to their CA1 area, and *in vitro* SUMOylation assay was performed 48 h later. Results revealed that Flag-MeCP2WT transfection increased the level of MeCP2 SUMOylation, but this effect was blocked by either Flag-MeCP2S421A or Flag-MeCP2T308A transfection (Fig. 3a). The quantified result is shown in the lower panel.

### MeCP2 SUMOylation is induced by NMDA and IGF-1 and CRF

We next examined whether MeCP2 SUMOylation is induced by neuronal activation using N-methyl-D-aspartate (NMDA) as a stimulus and whether the two phosphorylation mutants block NMDA-induced MeCP2 SUMOylation. Rats received Flag-vector, Flag-MeCP2WT, Flag-MeCP2S421A or Flag-MeCP2T308A transfection as described above. NMDA injection (2 μg μl^−1^) was made to the CA1 area of these rats 47 h after plasmid transfection. They were killed 1 h after NMDA injection, and their CA1 tissue was punched out for *in vitro* SUMOylation assay. Another group of rats receiving Flag-vector transfection and PBS injection served as the control. Results revealed that NMDA injection increased the level of MeCP2 SUMOylation. This effect was further enhanced by Flag-MeCP2WT transfection, but was abolished by Flag-MeCP2S421A and Flag-MeCP2T308A transfections (Fig. 3b). Consistent with previous reports that neuronal activation increases the phosphorylation level of MeCP2 at Ser-421 and Thr-308 (refs 19, 21), NMDA injection also increased the phosphorylation level of MeCP2 at Ser-421 (Fig. 3b). This effect was similarly enhanced by Flag-MeCP2WT transfection, but was blocked by Flag-MeCP2S421A transfection. It was unaffected by Flag-MeCP2T308A transfection (Fig. 3b). The quantified results are shown in the lower panel.

We next examined whether other stimuli may also affect MeCP2 SUMOylation. Insulin-like growth factor-1 (IGF-1) was suggested as a potential treatment for RTT patients^29^,^30^; here we examined whether MeCP2 SUMOylation may be a molecular target of IGF-1. Rats received PBS or IGF-1 (100 ng ml^−1^) injection to their CA1 area, and MeCP2 SUMOylation was determined 1 h later. Results revealed that IGF-1 significantly increased the level of MeCP2 SUMOylation. In addition, IGF-1 seemed to yield more sumo-bands, and this is evident by immunoblotting with both anti-MeCP2 (Fig. 3c, left panel) and anti-SUMO1 (Fig. 3c, right panel) antibodies. Meanwhile, IGF-1 increased the phosphorylation level of MeCP2 at Ser-421 (Fig. 3c, lower-left panel). There are other substances also known to enhance cognitive function and *Bdnf* gene expression, such as corticotropin-releasing factor (CRF)^31^,^32^ and dexamethasone (a synthetic glucocorticoid)^33^,^34^, and they may have the potential to ameliorate the RTT syndrome. Here we examined whether CRF and dexamethasone also increase MeCP2 SUMOylation. Rats received PBS or CRF (100 ng μl^−1^) injection to their CA1 area. They were killed 1 h later and their CA1 tissue was subjected to *in vitro* SUMOylation assay. In another experiment, rats received dimethylsulphoxide (DMSO) or dexamethasone (30 ng μl^−1^) injection in their CA1 area. They were killed 1 h later and their CA1 tissue was subjected to MeCP2 SUMOylation determination. Results revealed that CRF markedly increased the level of MeCP2 SUMOylation (Fig. 3d); however, dexamethasone did not produce the same effect (Fig. 3e).

### SUMOylation of MeCP2 decreases its interaction with CREB

**Figure 4:**
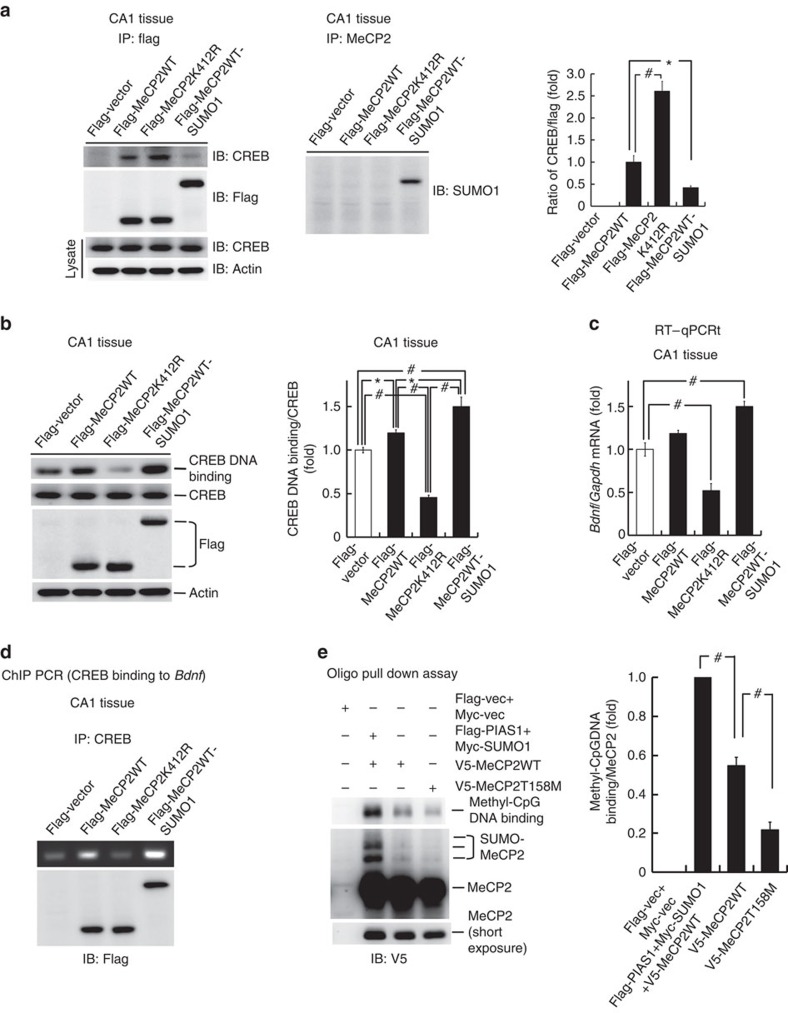
SUMOylation of MeCP2 decreases its interaction with CREB and increases CREB DNA binding and *Bdnf* gene expression and methyl-DNA binding. (**a**) Flag-vector, Flag-MeCP2WT, Flag-MeCP2K412R or Flag-MeCP2WT-SUMO1 fusion plasmid was transfected to rat CA1 area, and the association between MeCP2 and CREB was examined by co-IP using anti-Flag and anti-CREB antibodies. Plasmid transfection and expression was confirmed with western blot analysis using anti-Flag antibody (left panel). Cell lysates were also immunoprecipitated with anti-MeCP2 antibody and immunoblotted with anti-SUMO1 antibody showing the specific band of sumoylated MeCP2 (middle panel). The quantified result of co-IP is shown in the right panel (*n*=3 each group; F_3,8_=72.69, #*P*<0.001; *q*=11.99, #*P*<0.001, Flag-MeCP2K412R group versus Flag-MeCP2WT group; *q*=4.31, *P<0.05, Flag-MeCP2WT-SUMO1 group versus Flag-MeCP2WT group. (**b**) The same plasmids were transfected to the rat CA1 area, and CREB DNA-binding activity was determined using oligo pull-down assay. CREB expression was determined with western blot analysis. Plasmid transfection and expression was confirmed with western blot analysis using anti-Flag antibody (*n*=7 each group; F_3,24_=52.07, #*P*<0.001; *q*=3.26, *P<0.05, Flag-MeCP2WT group versus Flag-vector group; *q*=8.94, #*P*<0.001, Flag-MeCP2K412R group versus Flag-vector group; *q*=8.2, #*P*<0.001, Flag-MeCP2WT-SUMO1 group versus Flag-vector group; *q*=12.21, #*P*<0.001, Flag-MeCP2K412R group versus Flag-MeCP2WT group; *q*=4.93, *P<0.05, Flag-MeCP2WT-SUMO1 group versus Flag-MeCP2WT group; *q*=17.14, #*P*<0.001, Flag-MeCP2WT-SUMO1 group versus Flag-MeCP2K412R group). (**c**) The same plasmids were transfected to the rat CA1 area, and the *Bdnf* mRNA level was determined with RT–qPCR and quantified over the *Gapdh* mRNA level (*n*=6 each group; F_3,20_=38.45, #*P*<0.001; *q*=7.26, #*P*<0.001, Flag-MeCP2K412R group versus Flag-vector group; *q*=7.56, #*P*<0.001, Flag-MeCP2WT-SUMO1 group versus Flag-vector group. (**d**) The same plasmids were transfected to the rat CA1 area, and direct CREB binding to the *Bdnf* promoter was determined using chromatin IP assay. Plasmid transfection and expression was confirmed with western blot analysis using anti-Flag antibody. (**e**) V5-MeCP2T158M, V5-MeCP2WT alone or together with the Flag-PIAS1 and Myc-SUMO1 plasmids were co-transfected to HEK293T cells and cell lysates were subjected to methyl-DNA-binding assay and *in vitro* SUMOylation assay (*n*=3 each group; F_3,8_=230.02, #*P*<0.001; *q*=11.53, #*P*<0.001, V5-MeCP2T158M group versus V5-MeCP2WT group; *q*=15.75, #*P*<0.001, Flag-PIAS1+Myc-SUMO1+V5-MeCP2WT group versus V5-MeCP2WT group). One-way ANOVA followed by *post hoc* Newman–Keuls multiple comparisons. Data are expressed as mean±s.e.m.

In this experiment, we investigated the molecular events downstream of MeCP2 SUMOylation. Because *Bdnf* plays an important role in RTT and in mouse model of RTT^35^,^36^,^37^,^38^ and CREB binds to the *Bdnf* promoter directly^25^, we first investigated whether SUMOylation of MeCP2 alters its interaction with CREB. Rats received Flag-vector, Flag-MeCP2WT, Flag-MeCP2K412R or Flag-MeCP2WT-SUMO1 transfection in their CA1 area. They were killed 48 h later and their CA1 tissue was subjected to co-IP experiment. Results revealed that MeCP2 is associated with CREB; this association was increased by Flag-MeCP2K412R transfection and diminished by Flag-MeCP2WT-SUMO1 transfection (Fig. 4a, left panel). To further confirm that the higher molecular weight band indicates sumoylated MeCP2, cell lysates from the same groups were immunoprecipitated with anti-MeCP2 antibody and immunoblotted with anti-SUMO1 antibody. Result revealed that a specific band was observed at the same molecular weight in the Flag-MeCP2-SUMO1 group only (Fig. 4a, middle panel). The quantified result is shown in the right panel.

### MeCP2 SUMOylation increases CREB DNA binding in the brain

One possible explanation for the above result is that, on MeCP2 SUMOylation, CREB is released from the MeCP2 repressor complex and becomes more available for DNA binding and transcriptional regulation. This hypothesis was examined here. Flag-vector, Flag-MeCP2WT, Flag-MeCP2K412R and Flag-MeCP2WT-SUMO1 were transfected in the rat CA1 area. Animals were killed 48 h later and their CA1 tissue was subjected to CREB DNA-binding determination. Results revealed that Flag-MeCP2WT increased, whereas Flag-MeCP2K412R decreased CREB DNA binding when compared with the control group. Transfection of Flag-MeCP2WT-SUMO1 further increased CREB DNA binding compared with the Flag-MeCP2WT group (Fig. 4b, left panel). The expression level of CREB was not altered by these plasmid transfections.

### MeCP2 SUMOylation increases *Bdnf* gene expression

Because blockade of MeCP2 SUMOylation decreased CREB DNA binding and CREB directly binds to the *Bdnf* promoter^25^, we further examined whether SUMOylation of MeCP2 regulates *Bdnf* promoter activity. Different V5-tagged MeCP2 plasmids were transfected to Neuro2A cells with co-transfection of the *Bdnf* exon IV promoter construct and the *Renilla* luciferase-encoding construct (as an internal control). *Bdnf* promoter activity was determined 48 h later by reporter luciferase assay. Results showed that V5-MeCP2K412R decreased, whereas V5-MeCP2WT-SUMO1 increased *Bdnf* promoter activity (Supplementary Fig. 4a). We further examined the transfection efficiency in this experiment. mRFP-MeCP2WT was transfected to Neuro2A cells; total number of cells and the number of cells that showed red fluorescence were counted 48 h later. Results revealed that the transfection efficiency is ∼40% (Supplementary Fig. 4b).

Because CREB directly binds to the *Bdnf* promoter^25^ and MeCP2 SUMOylation regulates CREB DNA binding, it is conceivable that MeCP2 SUMOylation also regulates *Bdnf* mRNA expression. This hypothesis was examined here. Rats received Flag-vector, Flag-MeCP2WT, Flag-MeCP2K412R or Flag-MeCP2WT-SUMO1 transfections. They were killed 48 h later and their CA1 tissue was subjected to *Bdnf* mRNA determination. Results indicated that transfection of Flag-MeCP2WT increased the *Bdnf* mRNA level for ∼18%, but transfection of Flag-MeCP2K412R decreased the *Bdnf* mRNA level to ∼50% compared with the control group. Transfection of Flag-MeCP2WT-SUMO1 increased the *Bdnf* mRNA level for ∼50% (Fig. 4c). Because MeCP2 regulates the expression of a wide variety of genes, we further examined this issue by performing a cDNA microarray analysis. Results revealed that Flag-MeCP2K412R transfection altered the expression of ∼40,000 genes compared with Flag-MeCP2WT transfection. Among these genes, there were 1,368 genes that showed greater than or equal to twofold upregulation and 1,686 genes that showed greater than or equal to twofold downregulation. The ontology analysis of these genes is shown in Supplementary Table 1. To validate the result obtained from microarray analysis, we have selected three genes that were upregulated by Flag-MeCP2K412R transfection and three genes that were downregulated by Flag-MeCP2K412R transfection for further reverse transcriptase quantitative PCR (RT–qPCR) analysis. Results revealed that Flag-MeCP2K412R increased the mRNA level of *Olr640*, *Bco2* and *Mx1* genes for ∼9-, 4.3- and 3.8-fold, respectively; in addition, it decreased the mRNA level of *Igf2*, *Wnt6* and *Wnt5b* genes for ∼5-, 3.6- and 1.7-fold, respectively (Supplementary Fig. 5). Although the fold differences are not the same as those obtained from microarray analysis, the RT–qPCR results are in general consistent with the results obtained from microarray analysis. However, the *Bdnf* gene is not on the list of microarray analysis. It is not known whether the time point we selected for microarray analysis (48 h) is not best for detection of *Bdnf* mRNA alteration. Therefore, we have conducted a time course study to include the time intervals of 24, 36 and 48 h to further examine the effect of Flag-MeCP2K412R transfection on *Bdnf* mRNA expression in separate groups of animals. Results revealed that Flag-MeCP2K412R transfection significantly decreased the *Bdnf* mRNA level at all time intervals examined (Supplementary Fig. 6).

The above results showed that blockade of MeCP2 SUMOylation decreased CREB DNA binding and *Bdnf* mRNA expression; here we examined whether MeCP2 SUMOylation increases CREB binding to endogenous *Bdnf* promoter in the hippocampus. Flag-vector, Flag-MeCP2WT, Flag-MeCP2K412R and Flag-MeCP2WT-SUMO1 were transfected to the rat CA1 area. Animals were killed 48 h later and their CA1 tissue was subjected to chromatin immunoprecipitation (IP) assay. Results revealed that CREB directly binds to the endogenous *Bdnf* promoter. Blockade of MeCP2 SUMOylation by Flag-MeCP2K412R transfection decreased, whereas enhanced MeCP2 SUMOylation by Flag-MeCP2WT-SUMO1 transfection increased CREB binding to the *Bdnf* promoter when compared with the Flag-MeCP2WT group (Fig. 4d). In addition, the above results showed that MeCP2 interacts with CREB, and MeCP2 SUMOylation releases CREB and induces *Bdnf* gene expression in the hippocampus (Fig. 4a,c); here we further examined the interaction between MeCP2 and CREB on MeCP2 SUMOylation in HEK cells. V5-MeCP2WT or V5-MeCP2K412R was co-transfected with Flag-CREB to HEK293T cells and co-IP was carried out 48 h later. Results revealed that enhanced association between MeCP2 and CREB was observed when Flag-MeCP2K412R was transfected (compared with the Flag-MeCP2WT transfection group; Supplementary Fig. 7a). Similar results were obtained when the antibodies used for IP and immunoblotting exchanged (Supplementary Fig. 7b).

### MeCP2 SUMOylation increases its methyl-DNA binding

Several *MECP2* mutations identified in RTT patients show either altered interaction with other proteins or impaired methyl-DNA binding^10^,^11^. The above results showed that SUMOylation of MeCP2 alters its interaction with CREB; here we examined whether MeCP2 SUMOylation alters its methyl-DNA binding. V5-MeCP2WT was transfected to HEK293T cells with or without the co-transfection of Flag-PIAS1 and Myc-SUMO1. V5-MeCP2T158M was transfected as a negative control. Results revealed that MeCP2 methyl-DNA binding was observed when Flag-MeCP2WT was transfected, but co-transfection of Flag-PIAS1 and Myc-SUMO1 markedly increased MeCP2 methyl-DNA binding; it also increased MeCP2 SUMOylation (Fig. 4e). However, transfection of V5-MeCP2T158M almost completely abrogated MeCP2 methyl-DNA binding and MeCP2 SUMOylation (Fig. 4e).

### *MECP2* mutations in RTT show decreased MeCP2 SUMOylation

**Figure 5:**
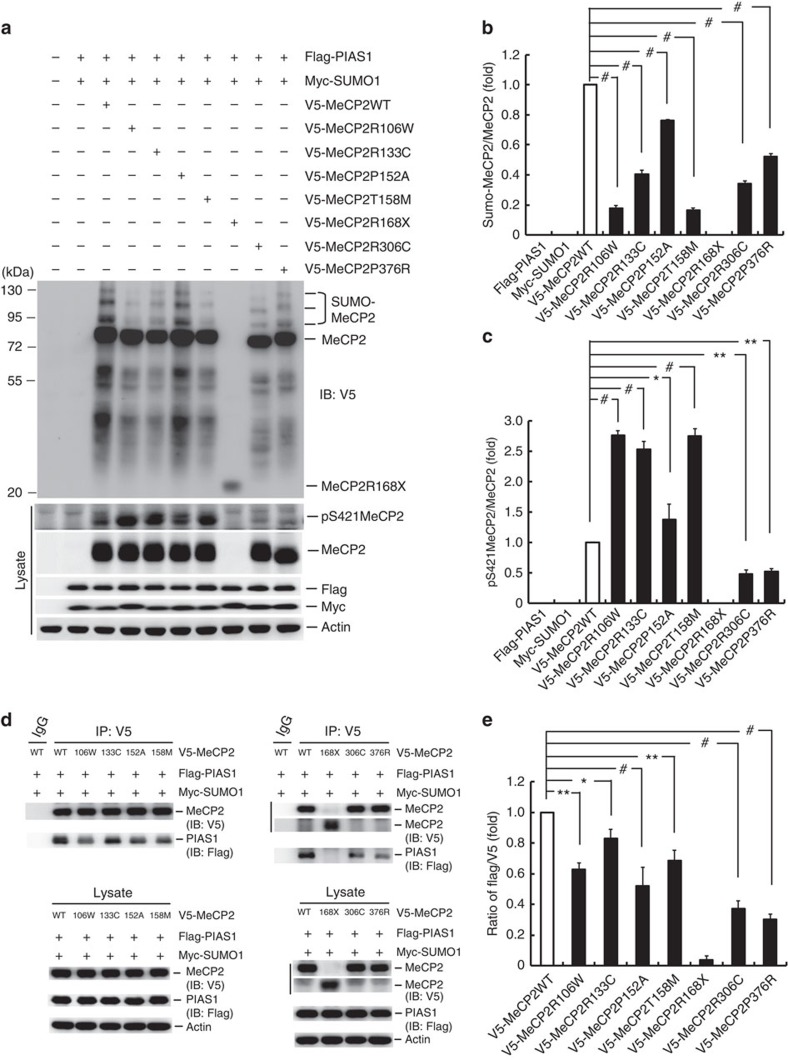
Several *MECP2* mutations identified in RTT patients show a decreased level of MeCP2 SUMOylation and decreased interaction with PIAS1. (**a**) V5-MeCP2WT plasmid or different V5-MeCP2-mutant plasmids associated with RTT were co-transfected with Flag-PIAS1 and Myc-SUMO1 plasmids to HEK293T cells and cell lysates were subjected to *in vitro* SUMOylation assay. The level of MeCP2 phosphorylation at Ser-421 was examined using the phospho-Ser-421 MeCP2 antibody. The lower band in lane 8 indicates the truncated MeCP2R168X protein. (**b**) The quantified result of MeCP2 SUMOylation is shown (*n*=4 each group; F_9,30_=579.1, #*P*<0.001; *q*=57.8, #*P*<0.001, V5-MeCP2R106W group versus V5-MeCP2WT group; *q*=41.81, #*P*<0.001, V5-MeCP2R133C group versus V5-MeCP2WT group; *q*=16.69, #*P*<0.001, V5-MeCP2P152A group versus V5-MeCP2WT group; *q*=58.68, #*P*<0.001, V5-MeCP2T158M group versus V5-MeCP2WT group; *q*=46.2, #*P*<0.001, V5-MeCP2R306C group versus V5-MeCP2WT group; *q*=33.56, #*P*<0.001, V5-MeCP2P376R group versus V5-MeCP2WT group, one-way ANOVA followed by *post hoc* Newman–Keuls multiple comparisons). (**c**) The quantified result of MeCP2 phosphorylation at Ser-421 is shown (*n*=3 each group; F_9,20_=119.55, #*P*<0.001; *q*=16.73, #*P*<0.001, V5-MeCP2R106W group versus V5-MeCP2WT group; *q*=14.48, #*P*<0.001, V5-MeCP2R133C group versus V5-MeCP2WT group; *q*=3.54, *P<0.05, V5-MeCP2P152A group versus V5-MeCP2WT group; *q*=16.63, #*P*<0.001, V5-MeCP2T158M group versus V5-MeCP2WT group; *q*=4.97, ^**^P<0.01, V5-MeCP2R306C group versus V5-MeCP2WT group; *q*=4.52, ^**^P<0.01, V5-MeCP2P376R group versus V5-MeCP2WT group, one-way ANOVA followed by *post hoc* Newman–Keuls multiple comparisons). (**d**) V5-MeCP2WT plasmid or V5-tagged individual MeCP2-mutant plasmid was transfected to HEK293T cells, and co-IP experiments were carried out with immunoprecipitation using anti-V5 antibody and immunoblotting using anti-Flag antibody. The expression level of MeCP2 (WT or mutant proteins) was examined with western blot analysis using anti-V5 antibody. (**e**) The quantified result of the association between PIAS1 and MeCP2 (or MeCP2-mutant proteins) is shown (*n*=3 each group; F_7,16_=27.47, #*P*<0.001; *q*=5.85, ^**^P<0.01, V5-MeCP2R106W group versus V5-MeCP2WT group; *q*=3.01, *P=0.05, V5-MeCP2R133C group versus V5-MeCP2WT group; *q*=8.46, #*P*<0.001, V5-MeCP2P152A group versus V5-MeCP2WT group; *q*=5.60, ^**^P<0.01, V5-MeCP2T158M group versus V5-MeCP2WT group; *q*=11.09, #*P*<0.001, V5-MeCP2R306C group versus V5-MeCP2WT group; *q*=10.88, #*P*<0.001, V5-MeCP2P376R group versus V5-MeCP2WT group, one-way ANOVA followed by *post hoc* Newman–Keuls multiple comparisons). Data are expressed as mean±s.e.m.

In this experiment, we examined whether *MECP2* mutations identified in RTT patients show abnormal MeCP2 SUMOylation. We first examined the level of MeCP2 SUMOylation in seven *MECP2* mutations that are most commonly seen in RTT patients^9^,^39^,^40^. Individual V5-tagged *MECP2*-mutant plasmid was transfected to HEK293T cells and *in vitro* SUMOylation assay was performed 48 h later. Results revealed that *MECP2* mutants R106W, R133C, P152A, T158M, R306C and P376R significantly decreased MeCP2 SUMOylation to different extent. R168X mutant did not show any MeCP2 SUMOylation because it is a truncated protein (Fig. 5a). The quantified result is shown in Fig. 5b. However, these mutations do not cover the residues that are sumoylated (Lys-363 and Lys-412) or phosphorylated (Thr-308 and Ser-421) that may regulate MeCP2 SUMOylation. To study why these mutants showed decreased SUMOylation, we examined whether they may alter the level of MeCP2 phosphorylation. Results indicated that only MeCP2R306C and MeCP2P376R showed decreased MeCP2 phosphorylation; other MeCP2 mutants showed increased MeCP2 phosphorylation (Fig. 5a). The quantified result is shown in Fig. 5c.

### *MECP2* mutations show decreased interaction with PIAS1

We further examined whether there is a decreased interaction between PIAS1 and these *MECP2* mutants that may explain the reduced MeCP2 SUMOylation seen in these mutants. Individual V5-tagged MeCP2-mutant plasmid was co-transfected with Flag-PIAS1 and Myc-SUMO1 to HEK293T cells and co-IP was carried out 48 h later. Results revealed that all the MeCP2 mutants showed reduced association with PIAS1 (Fig. 5d, upper panels). The quantified result is shown in Fig. 5e. The expression level of these transfected plasmids in cell lysates is similar (Fig. 5d, lower panels). We also examined the association between PIAS1 and MeCP2K223R. Result revealed that the interaction between PIAS1 and MeCP2K223R was not altered when compared with the MeCP2WT group (Supplementary Fig. 2b).

### MeCP2 SUMOylation rescues neural deficit in *Mecp2* cKO mice

**Figure 6:**
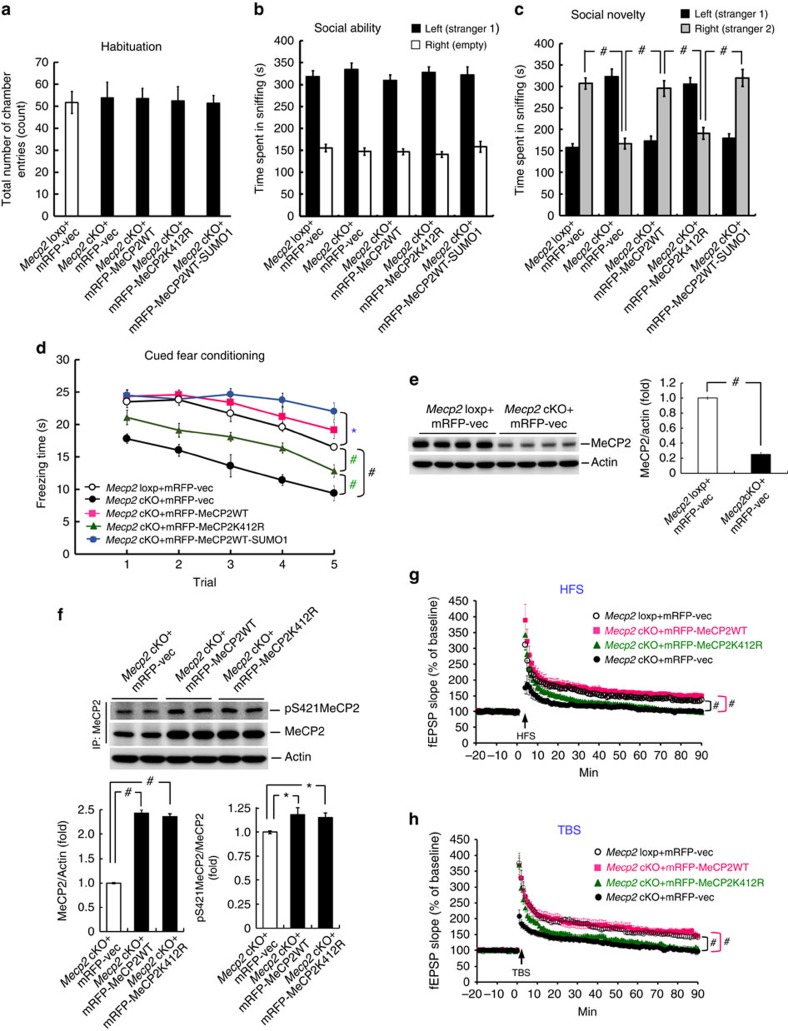
SUMOylation of MeCP2 rescues *Mecp2* cKO mice-induced behavioural and LTP deficits. *Mecp2* loxp and *Mecp2* cKO mice transducted with different MeCP2 lenti-mRFP vectors in their BLA area were subject to (**a**) motor activity test, (**b**) social ability test and (**c**) social novelty test 7–10 days later (*n*=10–11 each group; F_4,47_=19.87, #*P*<0.001 for sniffing to right stranger 2; *q*=8.55, *Mecp2* cKO+lenti-mRFP-vector group versus *Mecp2* loxp+lenti-mRFP-vector group; *q*=7.85, *Mecp2* cKO+lenti-mRFP-MeCP2WT group versus *Mecp2* cKO+lenti-mRFP-vector group; *q*=6.56, *Mecp2* cKO+lenti-mRFP-MeCP2K412R group versus *Mecp2* cKO+lenti-mRFP-MeCP2WT group; *q*=8.29, *Mecp2* cKO+lenti-mRFP-MeCP2WT-SUMO1 group versus *Mecp2* cKO+lenti-mRFP-MeCP2K412R group). (**d**) The same mice were subjected to cued fear-conditioning learning 7 days later (F_4,47_=37.17, #*P*<0.001; *q*=10.77, *Mecp2* cKO+lenti-mRFP-vector group versus *Mecp2* loxp+lenti-mRFP-vector group; *q*=5.27, *Mecp2* cKO+lenti-mRFP-MeCP2K412R group versus *Mecp2* loxp+lenti-mRFP-vector group; *q*=5.75, *Mecp2* cKO+lenti-mRFP-MeCP2K412R group versus *Mecp2* cKO+lenti-mRFP-vector group; *q*=4.07, *Mecp2* cKO+lenti-mRFP-MeCP2WT-SUMO1 group versus *Mecp2* loxp+lenti-mRFP-vector group). (**e**) The BLA tissues from *Mecp2* loxp+lenti-mRFP-vector and *Mecp2* cKO+lenti-mRFP-vector groups of mice were subjected to western blot analysis of MeCP2 expression (*t*_1,18_=20.99, #*P*<0.001). (**f**) The BLA tissue of mice from *Mecp2* cKO+lenti-mRFP vector, *Mecp2* cKO+lenti-mRFP-MeCP2WT and *Mecp2* cKO+lenti-mRFP-MeCP2K412R groups were subjected to western blot analysis of MeCP2 expression and MeCP2 phosphorylation at Ser-421 (for MeCP2 expression, F_2,28_=241.64, #*P*<0.001; *q*=27.46, *Mecp2* cKO+lenti-mRFP-MeCP2WT group versus *Mecp2* loxp+lenti-mRFP-vector group; *q*=26.58, *Mecp2* cKO+lenti-mRFP-MeCP2K412R group versus *Mecp2* loxp+lenti-mRFP-vector group. For pS421MeCP2, F_2,28_=3.72, *P<0.05; *q*=3.59, *Mecp2* cKO+lenti-mRFP-MeCP2WT group versus *Mecp2* loxp+lenti-mRFP-vector group; *q*=3.04, *Mecp2* cKO+lenti-mRFP-MeCP2K412R group versus *Mecp2* loxp+lenti-mRFP-vector group). (**g**) Aged female *Mecp2* cKO mice transducted with different MeCP2 lenti-mRFP vectors in their CA1 area were subjected to LTP recording 7 days later under HFS paradigm. The *Mecp2* loxp mice received lenti-mRFP vector transduction (*n*=5 each group; F_3,16_=14.36, #*P*<0.001; *q*=6.3, *Mecp2* cKO group versus *Mecp2* loxp group; *q*=8.45, *Mecp2* cKO+MeCP2WT group versus *Mecp2* cKO group; *q*=3.31, *Mecp2* cKO+MeCP2K412R group versus *Mecp2* cKO group for the first 10-min recording. (**h**) The same groups of mice were subjected to LTP recording under TBS paradigm (*n*=5 each group; F_3,16_=14.65, #*P*<0.001; *q*=7.08, *Mecp2* cKO group versus *Mecp2* loxp group; *q*=8.05, *Mecp2* cKO+MeCP2WT group versus *Mecp2* cKO group; *q*=4.69, *Mecp2* cKO+MeCP2K412R group versus *Mecp2* cKO group for the first 10-min recording). Arrow indicates delivery of HFS or TBS. One-way or two-way ANOVA and Newman–Keuls statistics. *P<0.05 and #*P*<0.001. Data are expressed as mean±s.e.m.

The above results revealed the mechanism of MeCP2 SUMOylation and its association with RTT; however, the functional significance of MeCP2 SUMOylation is not known. This issue was examined here. We have adopted the *Mecp2* loxp mice and recombinase *Cre* injection strategy to generate *Mecp2* cKO mice and the social interaction paradigm for the present experiments. Animals received lenti-mRFP-vector, lenti-mRFP-MeCP2WT vector, lenti-mRFP-MeCP2K412R vector or lenti-mRFP-MeCP2WT-SUMO1 fusion vector transduction to the basolateral amygdala (BLA) and were subjected to habituation and social interaction measures. The *Mecp2* loxp mice receiving lenti-mRFP-vector transduction served as the control. Anatomical localization of GFP-Cre expression and mRFP-MeCP2 expression in BLA neurons is shown by immunohistochemistry of green fluorescent protein (GFP; green) and monomeric red fluorescent protein (mRFP) (red), respectively (Supplementary Fig. 3c). Induction of MeCP2 SUMOylation on MeCP2 overexpression was confirmed in the BLA by *in vitro* SUMOylation assay (Supplementary Fig. 3d). Results revealed that the total number of chamber entries is similar among these five groups of animals (Fig. 6a), indicating that their locomotor activity level was similar. Result from social ability test revealed that animals in all the groups spent more time sniffing to stranger 1 than to the empty compartment, and their sniffing time to stranger 1 was also similar (Fig. 6b). Social novelty test was conducted 10 min after the social ability test. Results indicated that control animals spent more time sniffing to stranger 2 than to stranger 1, whereas the *Mecp2* cKO mice spent less time sniffing to stranger 2 compared with the control animals. However, for *Mecp2* cKO mice transducted with the lenti-mRFP-MeCP2WT vector, their sniffing time to stranger 2 was significantly increased compared with that of the *Mecp2* cKO mice. However, if the *Mecp2* cKO mice were transducted with the lenti-mRFP-MeCP2K412R vector, their sniffing time to stranger 2 was again decreased compared with that of *Mecp2* cKO mice transducted with the lenti-mRFP-MeCP2WT vector and is comparable to that of *Mecp2* cKO mice. When the *Mecp2* cKO mice were transducted with the lenti-mRFP-MeCP2WT-SUMO1 fusion vector, their sniffing time to stranger 2 was increased compared with that of *Mecp2* cKO mice transducted with the lenti-mRFP-MeCP2K412R vector and is comparable to that of control animals (Fig. 6c).

Because cognitive impairment is another behavioural deficit observed in RTT patients and in mouse model of RTT^6^,^13^, we further examined whether animals performed better in the social interaction task also showed better memory retention. The same animals were subjected to the cued fear-conditioning learning task 7 days after the social novelty test. Their memory retention was measured 24 h later. Results revealed that the *Mecp2* cKO mice showed impaired fear memory compared with the control animals. However, if the *Mecp2* cKO mice were transducted with the lenti-mRFP-MeCP2WT vector, their memory performance was significantly improved compared with the *Mecp2* cKO mice, and is comparable to that of control animals. However, if the *Mecp2* cKO mice were transducted with the lenti-mRFP-MeCP2K412R vector, their memory performance was worse than that of control animals, but was better than that of the *Mecp2* cKO mice. Further, when the *Mecp2* cKO mice were transducted with the lenti-mRFP-MeCP2WT-SUMO1 fusion vector, their memory performance was significantly improved compared with *Mecp2* cKO mice transducted with the lenti-mRFP-MeCP2K412R vector, and is comparable to that of *Mecp2* cKO mice transducted with the lenti-mRFP-MeCP2WT vector (Fig. 6d).

To demonstrate that Cre expression in the amygdala leads to successful depletion of MeCP2 expression, the BLA tissue of animals from the control group and *Mecp2* cKO group were subjected to western blot determination of MeCP2 expression. Results revealed that recombinase *Cre* transduction led to a significant decrease (∼75%) of MeCP2 expression in BLA neurons (Fig. 6e). The above results revealed that the *Mecp2* cKO mice transducted with the lenti-mRFP-MeCP2K412R vector showed better memory performance compared with *Mecp2* cKO mice. We suspected that the overexpressed MeCP2K412R may still be phosphorylated that leads to the signal transduction mediating fear memory formation. To test this hypothesis, animals from the *Mecp2* cKO+vector group, *Mecp2* cKO+MeCP2WT group and *Mecp2* cKO+MeCP2K412R group were killed after the fear memory test and their BLA tissue was subjected to western blot determination for pS421MeCP2 and MeCP2. Results revealed that the expression level of MeCP2 was increased to nearly 2.5-fold in both lenti-mRFP-MeCP2WT and lenti-mRFP-MeCP2K412R transduction groups. Yet, the phosphorylation level of MeCP2 was still increased for ∼16% in both groups (Fig. 6f). Because the same animals were subjected to both social interaction and fear-conditioning learning, we next examined whether there is a correlation between the performances of these two behaviours. The correlation between individual scores for the time spent sniffing to stranger 2 and individual scores of fear memory performance is shown in Supplementary Fig. 8a. If these individual scores were represented by the group mean values, the correlation is shown in Supplementary Fig. 8b. The correlation coefficient analysis revealed a significant correlation between these two measures.

Because Lys-223 was suggested as a candidate SUMO site on MeCP2 (ref. 27), we further examined the effect of MeCP2K223R on social interaction behaviour in rats. Animals received Flag-MeCP2WT or Flag-MeCP2K223R transfection to their BLA area, and their social interaction behaviours were measured 48 h later. Results revealed that MeCP2K223R transfection did not affect motor activity, social ability and social novelty performance in these animals (Supplementary Fig. 2c–e, respectively).

The above results showed that neuronal activation (NMDA injection) increased MeCP2 SUMOylation, and MeCP2 phosphorylation (at Ser-421 and Thr-308) facilitates MeCP2 SUMOylation (Fig. 3b); however, it is not known whether lack of SUMOylation affects MeCP2-mediated neuronal plasticity. This issue was examined here by adopting both the high-frequency stimulation (HFS) paradigm and theta-burst stimulation (TBS) paradigm of long-term potentiation (LTP) recording. The *Mecp2* cKO mice were transducted with lenti-mRFP-vector, lenti-mRFP-MeCP2WT vector or lenti-mRFP-MeCP2K412R vector to their CA1 area. The *Mecp2* loxp mice receiving mRFP-vector transduction served as the control. They were killed 7 days later and their hippocampal tissue slice was subjected to extracellular recording of field excitatory postsynaptic potential (fEPSP). Results revealed that when the HFS paradigm was used, the induction and expression of LTP was significantly impaired in *Mecp2* cKO mice compared with the *Mecp2* loxp mice. Overexpression of MeCP2WT in *Mecp*2 cKO mice rescued this LTP impairment, but overexpression of MeCP2K412R failed to do so. However, the early induction of LTP (first 10 min) was less affected in *Mecp2* cKO mice overexpressed with MeCP2K412R compared with that of the *Mecp2* loxp mice (Fig. 6g). Similar results were obtained with the TBS paradigm (Fig. 6h).

MeCP2 dosage in the brain is important for its function^41^. Reduction of MeCP2 for ∼40% in BLA is enough to cause behavioural deficits in mice^42^. Conversely, mice with MeCP2 overexpression for ⩾40% produce deficits in motor coordination, memory function and synaptic transmission, symptoms observed in MeCP2 duplication syndrome^43^. Here we examined the effect of different degrees of MeC2 depletion in BLA neurons on motor function and social interaction behaviour in rats. Animals received different concentrations of MeCP2 siRNA transfection to their BLA area (0, 4, 8 and 16 pmol). Social interaction behaviours were conducted 48 h later. Results revealed that all concentrations of MeCP2 siRNA examined did not affect animals' motor function (Supplementary Fig. 9a) and social ability (Supplementary Fig. 9b). However, MeCP2 siRNA produced a dose-dependent increase in the time spent sniffing to stranger 1, and a dose-dependent decrease in the time spent sniffing to stranger 2 during the social novelty test (Supplementary Fig. 9c). Western blot analysis revealed that MeCP2 siRNA transfection produced a dose-dependent decrease in MeCP2 expression in BLA neurons (Supplementary Fig. 9d).

## Discussion

Various mutations of the *MECP2* gene were identified in RTT patients. These mutations show either decreased methyl-DNA binding or abnormal interaction with other proteins; however, the molecular mechanism underlying these mutation-caused abnormal behaviours in RTT is less well known. In the present study, we have found that MeCP2 could be SUMO-modified by PIAS1 at Lys-412 and blockade of MeCP2 SUMOylation at this residue worsens behavioural deficits in *Mecp2* cKO mice resembling that of RTT patients. In addition to Lys-412, there are other possible SUMO acceptors on MeCP2, such as Lys-363, but blockade of MeCP2 SUMOylation at Lys-412 prevented MeCP2 SUMOylation at Lys-363 also. In examination of the relationship between MeCP2 SUMOylation and RTT, we have found a significant decrease in MeCP2 SUMOylation in several *MECP2* mutations identified in RTT. This result suggests that impaired MeCP2 SUMOylation may be a common mechanism underlying RTT. In examination of the molecular mechanism, we found that all the MeCP2-mutant proteins examined show decreased interaction with PIAS1. This may partially explain why decreased MeCP2 SUMOylation was observed with these mutations. However, why mutation at these residues leads to decreased interaction with PIAS1 is not known. It could be due to structural changes of these mutant proteins. Besides, we found that two of these mutant proteins also show decreased MeCP2 phosphorylation at Ser-421 (MeCP2R306C and MeCP2P376R). This also partially explains decreased MeCP2 SUMOylation with these two proteins because MeCP2 phosphorylation facilitates MeCP2 SUMOylation. However, all other MeCP2 mutations show an increased level of MeCP2 phosphorylation. It is possible that a compensation mechanism takes place because of decreased MeCP2 SUMOylation of these proteins.

**Figure 7:**
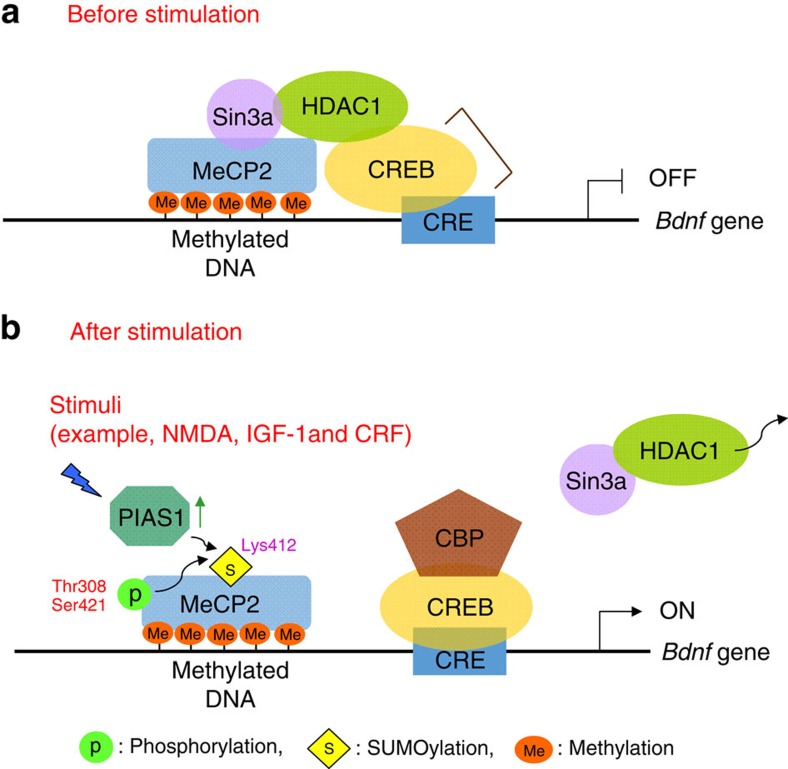
An illustration showing the relationship among MeCP2 SUMOylation, its association with CREB and other proteins in the repressor complex, CREB DNA binding to the *Bdnf* gene promoter and *Bdnf* gene expression. (**a**) Under physiological condition, MeCP2 binds to the methylated DNA and is associated with Sin3a, HDAC1 and CREB to form a repressor complex that suppresses CREB binding to the CRE element and inhibits *Bdnf* gene expression. (**b**) On stimulation, MeCP2 phosphorylation (at Thr-308 and Ser-421)-dependent MeCP2 SUMOylation takes place that enhances MeCP2 binding to methylated DNA, dissociates CREB from the repressor complex, which allows more CREB, in association with CBP, to bind to the CRE element on DNA promoter and increases *Bdnf* gene expression.

In the present study, we found that SUMOylation of MeCP2 increases CREB binding to the *Bdnf* promoter and *Bdnf* mRNA expression, and blockade of MeCP2 SUMOylation at Lys-412 increases the association between MeCP2 and CREB. On the basis of these results, we proposed a model in which sumoylated MeCP2 releases CREB from the repressor complex, allows more CREB to interact with its co-activator, such as CBP, and binds CRE element on the DNA promoter to regulate gene expression (Fig. 7). This model is consistent with the view that when MeCP2 interacts with HDAC and Sin3a, it functions as a transcriptional repressor^3^,^5^. Meanwhile, MeCP2K412R shows less CREB DNA binding. This may be due to more ‘free' CREB available that does not necessarily bind to endogenous DNA. Other than reduced interaction with CREB, sumoylated MeCP2 also shows an enhanced methyl-DNA binding. Therefore, the two mechanisms account for increased *Bdnf* mRNA expression on MeCP2 SUMOylation.

Accumulative lines of evidence have indicated that downregulation of BDNF is implicated in RTT^35^. For example, the BDNF level is lower in the brain of *Mecp2* knockout mice^36^,^37^ and deletion of *Bdnf* accelerates the progression of RTT-like symptoms in *Mecp2*-mutant mice^38^. An increase in the *Bdnf* level by fingolimod was found to improve symptoms in *Mecp2* KO mouse model of RTT^44^. Moreover, the *Bdnf* mRNA level was found to be decreased in the brain of two RTT patients^45^,^46^. In the present study, we found that Flag-MeCP2K412R transfection decreases, whereas Flag-MeCP2WT-SUMO1 transfection increases the *Bdnf* mRNA level in the rat hippocampus. These results are congruent with the above literature and suggest that SUMOylation of MeCP2 is an important mechanism regulating *Bdnf* gene expression associated with RTT. Because MeCP2 regulates the expression of a wide variety of genes^47^, we further examined this issue by performing a cDNA microarray analysis. Results revealed that Flag-MeCP2K412R transfection alters the expression of ∼40,000 genes and some of these genes are related to development, such as *Wnt6*. This result is consistent with the notion that RTT is a neurodevelopmental disease. It is also consistent with the view that MeCP2 may function as a regulator of brain development^7^.

In studying the mechanism of MeCP2 SUMOylation, we have found that MeCP2 phosphorylation (at Ser-421 and Thr-308) facilitates MeCP2 SUMOylation. We also found that NMDA injection increases the level of MeCP2 SUMOylation, but this effect was blocked by MeCP2S421A and MeCP2T308A transfections. Furthermore, we found that MeCP2 SUMOylation is enhanced on transfection of the constitutively active CaMKIIα (Supplementary Fig. 10a). This result is consistent with the report that MeCP2 phosphorylation at Ser-421 is CaMKII activity-dependent^19^. We have also examined the effect of other potential phosphorylation sites of MeCP2 on MeCP2 SUMOylation, including Ser-80, Ser-164, Ser-229 and Ser-399. Result revealed that none of these phosphorylation mutants alters the level of MeCP2 SUMOylation (Supplementary Fig. 10b). The present result also revealed that acute IGF-1 treatment increases the level of MeCP2 SUMOylation. In other studies, it was found that recombinant human IGF-1 treatment recovers behavioural impairment in *Mecp2*^-/y^ mouse model of RTT^48^, and repeated IGF-1 treatment improves cognitive function and social ability in a RTT patient^30^. These findings together support the notion that IGF-1 could be a potential therapeutic agent for RTT^29^. Our result also suggests that the potential therapeutic effect of IGF-1 is possibly mediated through enhanced MeCP2 SUMOylation. In addition, we found that CRF also increases MeCP2 SUMOylation. Together with the findings that CRF improves memory performance through increased *Bdnf* gene expression and that CRF induces long-lasting potentiation in hippocampal neurons *in vivo*^32^,^49^, these results suggest that CRF receptor-mediated signalling and perhaps corticosterone may also have a beneficial effect to RTT. However, glucocorticoid, which binds to the glucocorticoid receptor directly, did not affect MeCP2 SUMOylation. The molecular mechanism of CRF and the role of steroid hormones on MeCP2 SUMOylation are worth further investigating.

In examination of the functional significance of MeCP2 SUMOylation, we found that both *Mecp2* cKO and *Mecp2* cKO mice transducted with lenti-mRFP-MeCP2K412R showed impaired social novelty, memory performance and LTP; however, these deficits are rescued by transduction of lenti-mRFP-MeCP2WT and lenti-mRFP-MeCP2WT-SUMO1. These results together reveal the functional significance of MeCP2 SUMOylation, and they are consistent with the results that Flag-MeCP2K412R transfection decreased, whereas Flag-MeCP2WT-SUMO1 transfection increased the *Bdnf* mRNA level, and that BDNF plays an important role in memory function^50^. However, a more recent study indicated that *Mecp2* knock-in mice losing the function of MeCP2 phosphorylation at both Ser-421 and Ser-424 show an enhanced hippocampal LTP, learning and memory^51^. Our results seem incongruent with this finding, but they are consistent with another finding that MeCP2 phosphorylation at Ser-421 is induced by neuronal activation and it regulates *Bdn*f gene expression^19^. Other than the phosphorylation site examined in the above two studies being different (with and without Ser-424), another difference is that in the study showing enhanced LTP, genetically *Mecp2* knock-in mice were used, whereas in another study and our study, endogenous MeCP2 was knocked down and exogenous MeCP2S421 protein or MeCP2K412R mutant protein was overexpressed. There exists the possibility that a compensation mechanism may take place in *Mecp2*^S421A;S424A^ mice with their MeCP2 phosphorylation level at Thr-308 being higher, while we have found that MeCP2 phosphorylation at Thr-308 facilitates MeCP2 SUMOylation. It would be helpful to examine the MeCP2 phosphorylation level at Thr-308 and MeCP2 SUMOylation in *Mecp2*^S421A;S424A^ mice and that may partially explain the discrepancy.

*Mecp2* mutation and *Mecp2*-deficient mice often show locomotor deficits^14^,^16^,^52^; therefore, we also examined the locomotor activity in our animals. Result showed that the locomotor activity level is similar in all groups of animals, yet impairment in social novelty performance and fear memory was still observed in *Mecp2* cKO mice and *Mecp2* cKO mice transducted with lenti-mRFP-MeCP2K412R. This is probably because that the lentivectors were directly injected to the BLA area, but the BLA neurons do not play an important role in the regulation of motor function. In the study that *Mecp2*-deficient mice show motor dysfunction, significant reduction in the dopamine content was observed in the caudate–putamen^52^, while the caudate–putamen is important in the regulation of locomotor activity. MeCP2 is also implicated in the regulation of locomotor activity associated with psychostimulants. It was shown that the expression level of MeCP2 in the nucleus accumbens inversely regulates the reward properties of amphetamine, suggesting that MeCP2 controls the cellular and behavioural adaptations to this drug^53^. A more recent study indicates that MeCP2 phosphorylation at Ser-421 may contribute to the additive behaviours induced by these psychostimulants because the MeCP2 S421A knock-in mice show an increased excitability of medium spiny neurons in the nucleus accumbens and reduced threshold for behavioural adaptation to these drugs^54^. These results suggest that MeCP2 phosphorylation at Ser-421 limits the neural plasticity in the mesolimbic–cortical circuit in response to chronic psychostimulant treatment.

The PIAS protein family consists of different members of the PIAS protein, including PIAS1, PIAS2, PIAS3 and PIAS4 (ref. 55). The reason we have chosen PIAS1 for the present study is because that PIAS1 has been studied the most and PIAS1 expression is associated with neuronal activation^24^. However, the present findings do not exclude the possibility that other PIAS family proteins also SUMO-modify MeCP2. In addition, Lys-412 is not one of the candidate SUMO sites identified using the LC-MS analysis. A possible explanation is because that trypsin was used to cut the lysine residues on MeCP2 for LC-MS analysis and the length of a.a. ranging from 7 to 20 would be most ideal for LC-MS detection of candidate residues^56^. However, the lysine residue nearest to Lys-412 on MeCP2 is located at Lys-377, which yields a peptide of 36 a.a. in length after trypsin cutting. A peptide of this length is easy to be fragmented and makes the LC-MS analysis less reliable. However, other possibilities also remain.

In summary, we have presently found that MeCP2 could be SUMO-modified by PIAS1 at Lys-412. MeCP2 SUMOylation is neuronal activity-dependent and MeCP2 phosphorylation (at Ser-421 and Thr-308) facilitates MeCP2 SUMOylation. Several *MECP2* mutations identified in RTT patients show a decreased level of MeCP2 SUMOylation and reduced interaction with PIAS1. Furthermore, enhanced MeCP2 SUMOylation decreases its association with CREB, increases CREB DNA binding and *Bdnf* mRNA expression in the rat brain. It also rescues the behavioural deficits of *Mecp2* cKO mice. Moreover, *Mecp2* cKO mice show an impaired LTP relative to *Mecp2* loxp control mice. Overexpression of the MeCP2WT protein in *Mecp2* cKO mice rescues this impairment; however, overexpression of the MeCP2K412R mutant protein in *Mecp2* cKO mice fails to do so. Stimuli such as IGF-1 and CRF that markedly increase the level of MeCP2 SUMOylation without altering the MeCP2 protein level may have therapeutic potential against RTT and other autism-like syndrome.

## Methods

### Animals

Adult male (2–3 months) and aged female (6–8 months) *Mecp2*^loxp^ mice were used in this study. They were purchased from Jackson Laboratory (Bar Harbor, ME, USA; strain name: B6.129P2-*Mecp2*tm1Bird/J, stock number: 006847), bred and mated at the Animal Facility of the Institute of Biomedical Sciences (IBMS), Academia Sinica in Taiwan. Adult male Sprague–Dawley rats (250–350 g), bred at the Animal Facility of IBMS, were also used in the present study. All the animals were housed and maintained on a 12/12 h light/dark cycle (light on at 6:30 am) with food and water continuously available. Animals were randomly divided into different experimental groups. Experimental procedures followed the guidelines and ethical regulations of Animal Use and Care of the National Institute of Health and were approved by the Animal Committee of IBMS, Academia Sinica.

### Hippocampal lysate and cell lysate preparation

Animals were killed by decapitation, and their hippocampal tissue was dissected out. Rat hippocampal tissue was lysed by brief sonication in lysis buffer containing 50 mM Tris-HCl (pH 7.4), 150 mM NaCl, 2 mM EDTA, 1% IGEPAL CA-630, 1 mM phenylmethylsulfonyl fluoride (PMSF), 20 μg ml^−1^ pepstatin A, 20 μg ml^−1^ leupeptin, 20 μg ml^−1^ aprotinin, 50 mM NaF and 1 mM Na_3_VO_4_. The primary neuron cell lysate and HEK293T cell lysate were prepared in 1 ml of lysis buffer containing 20 mM Tris (pH 7.4), 150 mM NaCl, 1 mM MgCl_2_, 1% IGEPAL CA-630, 10% glycerol, 1 mM dithiothreitol (DTT), 50 mM β-glycerophosphate, 50 mM NaF, 10 μg ml^−1^ PMSF, 4 μg ml^−1^ aprotinin, 4 μg ml^−1^ leupeptin and 4 μg ml^−1^ pepstatin.

### IP and western blot

For IP PIAS1, MeCP2, Flag, V5 and Myc, the clarified lysate (0.5 mg) was immunoprecipitated overnight with 0.5 μl of anti-PIAS1 antibody (Catalogue No. 2474-1, Epitomics, Burlingame, CA), 2 μl of anti-MeCP2 antibody (Catalogue No. 3456, Cell Signaling, Danvers, MA), 2 μl of anti-Flag M2 antibody (Catalogue No. F1804, Sigma-Aldrich, St Louis, MO), 1 μl of anti-V5 antibody (Catalogue No. MCA2895, AbD Serotec, Kidlington, UK) and 2 μl of anti-Myc antibody (Catalogue No. 05-419, Millipore, Bedford, MA) at 4 °C for. Twenty microlitres of rabbit or mouse IgG were used in the control group. The protein A or G magnetic beads (30 ml, 50% slurry, GE Healthcare, Barrington, IL) were added to the IP reaction product to catch the immune complex at 4 °C for 3 h. The immune complexes on beads were washed three times with washing buffer containing 20 mM HEPES (pH 7.4), 150 mM NaCl, 1 mM EDTA, 1% IGEPAL CA-630, 1 mM DTT, 50 mM β-glycerophosphate, 50 mM NaF, 10 mg ml^−1^ PMSF, 4 μg ml^−1^ aprotinin, 4 μg ml^−1^ leupeptin and 4 μg ml^−1^ pepstatin, and were subjected to 8% SDS–PAGE, followed by transferring onto the nitrocellulose membrane (GE Healthcare). Western blot was conducted using the following antibodies: rabbit anti-PIAS1 (1:10,000, Catalogue No. 2474-1, Epitomics), anti-MeCP2 (1:2,000, Catalogue No. 3456, Cell Signaling), anti-phospho-Ser421MeCP2 (1:1,000, Catalogue No. AP3693a, ABGENT, San Diego, CA), anti-SUMO1 (1:4000, Catalogue No. 40120 SUMOlink kit, Active Motif, Carlsbad, CA), anti-ubiquitin (1:3,000, Catalogue No. 3936, Cell Signaling), anti-Flag M2 (1:5,000, Catalogue No. F1804, Sigma-Aldrich), anti-V5 (1:8,000, Catalogue No. MCA2895, AbD Serotec), anti-His (1:5,000, Catalogue No. OB05, Millipore), anti-GST (1:5,000, Catalogue No. 110736, GeneTex, San Antonio, TX), anti-RFP (1:2,000, Catalogue No. 600-401-379, Rockland, Gilbertsville, PA) and anti-actin (1:200,000, Catalogue No. MAB1501, Millipore). The secondary antibody used was horseradish peroxidase (HRP)-conjugated goat-anti-rabbit IgG antibody or goat-anti-mouse IgG antibody (Chemicon). Membrane was developed by reacting with chemiluminescent HRP substrate (Millipore) and exposed to the LAS-3000 image system (Fujifilm, Tokyo, Japan) for visualization of protein bands. The protein bands were quantified by using the NIH Image J Software.

### Plasmid construction and DNA transfection

For construction of the V5-tagged MeCP2 plasmid, full-length *Mecp2* was cloned by amplifying the rat hippocampal *Mecp2* cDNA (accession # NM_022673) with primers 5′- ATTTGCGGCCGCCACCATGGTAGCTGGGATGTTAG -3′ and 5′- TAACCGCGGGCTAACTCTCTCGGTCAC -3′. The PCR product was subcloned between the NotI and SacII sites of the mammalian expression vector pcDNA3.1-V5-His. *Mecp2*-mutant plasmids were generated using the QuickChange Site-Directed Mutagenesis Kit (Stratagene, La Jolla, CA). For construction of the mRFP-tagged *Mecp2* plasmid, wild-type *Mecp2*, *Mecp2K412R*, *Mecp2WT-SUMO1* and *Mecp2WT-SUMO1* fusion plasmids were subcloned into the pmRFP expression vector (Addgene plasmid #13990). For construction of the Flag-tagged *Pias1* plasmid, the procedure used was the same as that described previously^24^. HEK293T and Neuro2A cells were maintained in DMEM containing 10% fetal bovine serum and incubated at 37 °C in a humidified atmosphere with 5% CO_2_ as described previously^57^. Transfection was made by using the Lipofectamine 2000 reagent (Invitrogen, Carlsbad, CA) in 12-well culture plates according to the manufacturer's instructions.

### Lentiviral vector construction and preparation

For construction of mRFP, *mRFP-Mecp2WT*, *mRFP-Mecp2K412R* and *mRFP-Mecp2WT-SUMO1* lentivitral vectors, full-length mRFP-MeCP2WT, mRFP-MeCP2K412R and mRFP-MeCP2WT-SUMO1 fusion plasmids were subcloned into the lentiviral vector pLenti-Tri-cistronic (ABM, Richmond, BC, Canada) by amplifying different pmRFP-MeCP2-related non-viral constructs with different primers. The forward primer used for these five constructs is the same and it is 5′- ATCGGGATCCGCCACCATGGCCTCCTCCGAGGAC -3′. The reverse primer for *mRFP* vector is 5′- ATCGCCTAGGTTAGGCGCCGGTGGAGTGGCG -3′ and that for *mRFP-Mecp2WT* and *mRFP-Mecp2K412R* is 5′- ATCGCCTAGGTCAGCTAACTCTCTCGGTCACGGG -3′. The reverse primer used for *mRFP-Mecp2-SUMO1* and *mRFP-Mecp2WT-SUMO1* is 5′- ATCGCCTAGGCTAAACCGTCGAGTGACCCCCCG -3′. These PCR products were subcloned between BamHI and AvrII sites of the lentiviral vector. For construction of GFP-2A-NLS-Cre lentiviral vector, full-length Cre recombinase cDNA was added with nuclear localization signal (NLS) using PCR amplification and cloned into pLenti-Tri-cistronic (ABM) to obtain a bicistronic vector expressing both GFP and NLS-Cre. The primers used for Cre vector are 5′- ATCGGAATTCCCAAAGAAGAAGAGAAAGGTTATGTCCAATTTACTGACC -3′ (forward) and 5′- ATCGGCGGCCGCCTAATCGCCATCTTCCAG -3′ (reverse). The PCR product was subcloned between the EcoRI and NotI sites of the lentiviral vector pLenti-Tri-cistronic (ABM). The GFP construct was cloned by amplifying the *GFP* gene from pLenti-CMV-GFP-2A-Puro-Blank (ABM) and was subcloned into the pLenti-Tri-cistronic vector between ScaI and KpnI sites, upstream of the 2A peptide (a self-processing viral peptide bridge) and Nls-Cre sequences. The primers used for GFP vector are 5′- ATCGAGTACTGCCACCATGGAGATCGAGTGCCGCATC -3′ (forward) and 5′- ATCGGGTACCGGCGAAGGCGATGGGGGTC -3′ (reverse). For lentivirus packaging, HEK293LTV cells (Cell Biolabs, San Diego, CA) were transfected with 1.5 μg of psPAX2 (Addgene plasmid #12260), 0.5 μg of pMD2.G (Addgene plasmid #12259) and 2 μg of pLenti-GFP-2A-Nls-Cre, mRFP, *mRFP-Mecp2*, *mRFP-Mecp2K412R*, *mRFP-Mecp2WT-SUMO1* or 2 μg of pLenti-CMV-GFP-2A-Puro-Blank (ABM) coding for GFP as control using 10 μl of Lipofectamine 2000 (Invitrogen) in a six-well cell culture dish. Lentiviral particles were collected using the speedy lentivirus purification solution (ABM) according to the manufacturer's protocols. Cell culture medium containing lentiviral particles can be harvested for two to three times at 12-h interval until 36 h after transfection, and it was kept at 4 °C for the collecting period. The collected culture medium was further clarified by centrifugation at 2,500*g* for 10 min and filtrated through a 0.45-μm syringe filter. The speedy lentivirus purification solution (ABM) was added into filtrated supernatant (1:9, v/v) containing lentiviral particles and mixed thoroughly by inversion. The lentiviral supernatant was centrifuged at 5,000*g* at 4 °C for 10 min. Supernatant was then discarded and the viral pellet was resuspended in ice-cold PBS. After titration, the viral stock was stored at −80 °C in aliquots. The lentivirus titre was determined with the lentivirus qPCR Titer Kit (ABM) according to the manufacturer's protocols (ABM).

### *In vitro* SUMOylation assay

*In vitro* SUMOylation assay was performed by using the SUMOlink kit according to the manufacturer's instructions (Active Motif). Recombinant GST-tagged MeCP2 protein (Prospec, East Brunswick, NJ), His-tagged PIAS1 protein purified from His-bind resin (Novagen, WI) and GST-tagged SENP1 protein (Enzo, Ann Arbor, MI) were used for assays at 30 °C for 3 h and then boiled in Laemmli sample buffer at 95 °C for 10 min. The *in vitro* SUMOylation product was subjected to 8% SDS–PAGE followed by transferring onto the nitrocellulose membrane. The membrane was immunoblotted with anti-His and anti-GST antibodies.

### *In vitro* SUMOylation assay for CA1 tissue

Hippocampal CA1 tissue lysate was prepared in the same way as that prepared for western blot. For IP MeCP2, the clarified lysate (0.5 mg) was immunoprecipitated with 3 μl of anti-MeCP2 antibody (Catalogue No. 3456, Cell Signaling) at 41 °C overnight. The protein A agarose beads (30 ml, 50% slurry, GE Healthcare) were added to the IP reaction product to catch the immune complex at 41 °C for 3 h. The immune complexes on beads were washed three times with washing buffer containing 20 mM HEPES (pH 7.4), 150 mM NaCl, 1 mM EDTA, 1% IGEPAL CA-630, 1 mM DTT, 50 mM β-glycerophosphate, 50 mM NaF, 10 mg ml^−1^ PMSF, 4 mg ml^−1^ aprotinin, 4 mg ml^−1^ leupeptin and 4 mg ml^−1^ pepstatin and were subjected to *in vitro* SUMOylation reaction with the addition of recombinant PIAS1 protein (3 μl, Catalogue No. BML-UW9960, Enzo Life Sciences, Farmingdale, NY), E1 (1 μl), E2 (1 μl) and the SUMO1 (0.5 μl) proteins provided in the kit. *In vitro* SUMOylation assay was performed using the SUMO linkTM kit according to the manufacturer's instructions (Active Motif) and boiled in Laemmli sample buffer at 95 °C for 10 min. The *in vitro* SUMOylation product was subjected to 8% SDS–PAGE followed by transferring on the polyvinylidene difluoride (PVDF) membrane (Millipore). The membrane was immunoblotted with anti-MeCP2 antibody (1:1,000, Catalogue No. 3456, Cell Signaling) or anti-SUMO1 antibody (1:4,000, Catalogue No. 40120, Active Motif). For determination of endogenous MeCP2 SUMOylation after PIAS1 siRNA transfection, no E1, E2, SUMO1 and PIAS1 proteins were added to the IP reaction product. The remaining procedures were the same as those for carrying out the *in vitro* SUMOylation assay.

### Pull-down assay for MeCP2 methyl-DNA binding

DNA oligonucleotides containing CpG element from the *Bdnf* gene (underlined) (5′- CAATGCCCTGGAACGGAATTCTTCTAATAAAAGATGTATCATTTTAAATGC -3′) were conjugated with a 5′ biotin on the sense strand. DNA oligo sequence was synthesized at −125 base pairs from the transcription start site of *Bdnf* exon1. Both complementary oligonucleotides were annealed according to the procedure described previously^24^. The resulting DNA oligos were methylated with SssI methylase (NEB, Ipswich, MA) according to the recommended protocol for methylation of DNA (NEB). For the MeCP2 methyl-DNA-binding assay, the lysate (0.4 mg) from the HEK293T cell transfected with V5-MeCP2WT or V5-MeCP2T158M plasmid was added with 6 μl duplex oligonucleotides (100 μM) and poly dI–dC (1 μg ml^−1^, GE Healthcare) at 4 °C for overnight. The streptavidin agarose beads (10 μl, Sigma) were added to the pull-down reaction product to catch the MeCP2–DNA oligonucleotide complex at 4 °C for 3 h. The pull-down reaction complexes on beads were then washed three times with washing buffer containing 20 mM HEPES (pH 7.4), 150 mM NaCl, 1 mM EDTA, 1% IGEPAL CA-630, 1 mM DTT, 50 mM β-glycerophosphate, 50 mM NaF, 10 μg ml^−1^ PMSF, 4 μg ml^−1^ aprotinin, 4 μg ml^−1^ leupeptin and 4 μg ml^−1^ pepstatin, and then boiled in Laemmli sample buffer at 95 °C for 10 min. For the analysis of MeCP2 methyl-DNA-binding activity, the pull-down assay product was subjected to 8% SDS–PAGE, followed by transferring onto the PVDF membrane (Millipore), and was immunoblotted with anti-MeCP2 antibody (1:2,000, Catalogue No. 3456, Cell Signaling).

### Pull-down assay for CREB DNA-binding activity

DNA oligonucleotides containing two CRE elements (underlined) (5′- AGAGATTGCCTGACGTCAGAGAGCTAGGATTGCCTGACGTCAGAGAGCTAG -3′ for the sense strand and 5′- CTAGCTCTCTGACGTCAGGCAATCCTAGCTCTCTGACGTCAGGCAATCTCT -3′ for the antisense strand) were conjugated with a 5′ biotin on the sense strand according to the method described elsewhere^58^. Both complementary oligonucleotides were resuspended in the annealing buffer (10 mM Tris (pH 8.0), 50 mM NaCl, 1 mM EDTA). For annealing the sense and antisense oligonucleotides, 10 μl each of the complementary oligonucleotides together with 80 μl of the annealing buffer were mixed in a 0.5-ml microtube and the tube was placed in a heating block at 90 °C. The heating block was allowed to gradually cool down to room temperature and stored on ice or at −20 °C until use. For the CREB pull-down assay, the clarified hippocampal CA1 tissue lysate (0.4 mg) was added with 6-μl duplex oligonucleotides (100 μM) and poly dI–dC (1 μg ml^−1^, GE Healthcare) at 4 °C overnight. The streptavidin agarose beads (10 μl, Sigma-Aldrich) were added to the pull-down reaction product to catch the CREB DNA oligonucleotide complex at 4 °C for 3 h. The pull-down reaction complex on beads was then washed three times with PBS and boiled in Laemmli sample buffer at 95 °C for 10 min. For analysis of CREB DNA-binding activity, the pull-down assay product was subjected to 8% SDS–PAGE followed by transferring onto the PVDF membrane and immunoblotted with anti-CREB antibody (1:2,000, Catalogue No. 9197, Cell Signaling).

### RT–qPCR

Total RNA was isolated from 20 mg of hippocampal CA1 tissue using RNeasy Mini Kit (Qiagen, Germantown, MD) according to the manufacturer's instructions. The RNA samples were resuspended in nuclease-free water and quantified spectrophotometrically at 260 nm. All RNA samples had an *A*260:*A*280 value between 1.8 and 2.0. cDNA synthesis was carried out by using the QuantiTect Reverse Transcription Kit (Qiagen) according to the manufacturer's protocols. The cDNA stock was stored at −20 °C. Quantitative PCR for *Bdnf* and the endogenous control gene glyceraldehyde 3-phosphate dehydrogenase (*Gapdh*) was carried out by using the iQ SYBR Green Supermix (Bio-Rad). The primer sequences for *Gapdh* were as follows: 5′- GGCAAGTTCAATGGCACAGT -3′ (forward) and 5′- TGGTGAAGACGCCAGTAGACTC -3′ (reverse). The primer sequences for *Bdnf* were: 5′- CTAGGACTGGAAGTGGAAA -3′ (forward) and 5′- ATTTCATGCTAGCTCGCCG -3′ (reverse). Amplification was performed by using the Rotor-Gene Q Real Time PCR system (Qiagen), and the reaction condition followed the manufacturer's protocols. The thermal cycler protocol used is as follows: 95 °C for 10 min, 95 °C for 10 s and 60 °C for 30 s (40 cycles). The cycle threshold (Ct) values and related data were analysed by using the Rotor-Gene Q Real Time PCR System Software (Qiagen). The expression level of *Bdnf* was normalized with that of *Gapdh*. The relative expression levels (in fold) were determined by using the 2-^(ΔΔCt)^ method^59^.

### Extracellular field potentiation recording

*Mecp2* cKO mice overexpressed with lenti-mRFP vector, lenti-mRFP-MeCP2WT vector or lenti-mRFP-MeCP2K412R vector in their CA1 area were used for electrophysiological recording. Animals were killed and their brain slices were transferred to an immersion-type recording chamber and perfused with artificial cerebral spinal fluid (ACSF) containing 100 μM picrotoxin at a rate of 2 ml min^−1^ at room temperature. An incision was made between the CA1 and CA3 areas to remove afferent input from CA3. For the extracellular field potential recording, a glass pipette filled with 3 M NaCl was positioned in the CA1 stratum radiatum area to record the fEPSP. Bipolar stainless steel stimulating electrodes (Frederick Haer Company, Bowdoin, ME) were placed in the striatum radiatum to stimulate the Schaffer collateral pathway. Stable baseline fEPSP activity was recorded by applying a short-duration current stimulation pulse (∼40 μs) at a predetermined intensity every 15 s for at least 20 min. LTP was then induced by using both the HFS and TBS paradigms according to that described previously^18^. For HFS, LTP was induced by delivering two 100-Hz tetani (1 s) with an intertetanus interval of 20 s. For TBS, LTP was induced by delivering three trains of TBS. Each train consisted of 10 sets of bursts (four stimuli, 100 Hz) with an interburst interval of 200 ms. The interval between each stimulus train is 20 s.

### Drugs

NMDA was purchased from Tocris Bioscience (St Louis, MO, USA). IGF-1, CRF and dexamethasone were purchased from Sigma-Aldrich. NMDA, IGF-1 and CRF were dissolved in PBS immediately before use. Dexamethasone was dissolved in DMSO before injection.

### Intrahippocampal transfection and injection

Rats were anaesthetized with pentobarbital (40 mg kg^−1^) and subjected to stereotaxic surgery. Two 23-gauge, stainless steel, thin-wall cannulae were implanted bilaterally to the CA1 area of the rat brain at the following coordinates: 3.5 mm posterior to the bregma, ±2.5 mm lateral to the midline and 3.4 mm ventral to the skull surface. After recovery from the surgery, NMDA (2 μg μl^−1^), IGF-1 (100 ng ml^−1^), CRF (100 ng μl^−1^) and dexamethasone (30 ng μl^−1^) were directly injected to the CA1 area at a rate of 0.1 μl min^−1^. A total of 0.7 μl was injected to each side. For transient *Mecp2* plasmid DNA transfection, 0.7 μl the plasmid DNA complex (1.5 μg μl^−1^) was injected directly to the CA1 area bilaterally in the rat brain using the non-viral transfection agent polyethyleneimine (PEI)^60^, which we have previously demonstrated that it does not produce toxicity to hippocampal neurons^61^. Before injection, plasmid DNA was diluted in 5% glucose to a stock concentration of 2.77 μg μl^−1^. Branched PEI of 25 kDa (Sigma) was diluted to 0.1 M concentration in 5% glucose and added to the DNA solution. Immediately before injection, 0.1 M PEI was added to reach a ratio of PEI nitrogen per DNA phosphate of 10. The mixture was subjected to vortex for 30 s and was allowed to equilibrate for 15 min. For siRNA injection, 0.7 μl of PIAS1 siRNA (8 pmol) or control siRNA was transfected to the CA1 area bilaterally in the rat brain also using the transfection agent PEI. The sense and antisense sequences used for PIAS1 siRNA were adopted from those of a previous study^24^. The sequence for sense strand is 5′- UCCGGAUCAUUCUAGAGCUtt -3′ and that for antisense strand is 5′- AGCUCUAGAAUGAUCCGGAtt -3′. The Silencer Negative Control number 1 siRNA was used as a control. They both were synthesized from Ambion (Austin, TX). The inner diameter of the injection needle is 0.31 mm and the wall thickness of the injection needle is 0.12 mm. The injection needle was left in place for 5 min to limit the diffusion of injected agent. Animals were killed at different time points after drug injection, plasmid and siRNA transfection. Their brains were removed and cut with a brain slicer. Their CA1 tissue was further punched out by using a stainless punch with 2 mm inner diameter. Tissues were frozen at −80 °C until biochemical experimentation.

### Intra-BLA injection of lentiviral vectors

Mice were anaesthetized with pentobarbital (40 mg kg^−1^, intraperitoneally (i.p.)) and subjected to stereotaxic surgery without cannulation. Lentiviral vectors were directly injected to the BLA after the animals recovered from the surgery. The coordinates for the BLA are as follows: 0.5 mm posterior to the bregma, ±3.3 mm lateral to the midline and 4.8 mm ventral to the skull surface. We have adopted a replacement assay by co-infecting the floxed mice with both a lentivirus encoding Cre and a lentivirus encoding different forms of MeCP2 simultaneously. Similar approach has been adopted by other studies using mixed lentiviral vector transduction to primary hippocampal neurons of mice^62^. The recombinase *Cre* lentivector at a titre of 2 × 10^8^ per ml (diluted in PBS) was mixed with different mRFP-*Mecp2* lentivectors also at the titre of 2 × 10^8^ per ml (diluted in PBS) for each *Mecp2* vector at a 1:1 volume immediately before injection. A volume of 0.25 μl was injected to each side of BLA. The infusion rate was 0.1 μl min^−1^. Social interaction behaviours were measured 7–10 days after lentiviral vector transduction. Mice were killed after the fear-conditioning test. Their brains were removed and the BLA tissue was punched out as that described for the CA1 tissue. Tissues were also stored in a −80°C freezer until further experimentation.

### Three-chamber social ability and social novelty measures

Social ability and social novelty measures were conducted in a three-chamber cage at the following specifications: 60 × 40 × 22 cm (*L* × *W* × *H*). The procedures used for these measures were adopted from those of a previous study^63^. The chamber is divided into three compartments, with the left and right compartments being 21 cm in length and the middle compartment being 18 cm in length. There were two additional cylinder chambers with 15 cm in height and 10 cm in diameter placed in both the left and right compartments. During the social ability test, a stranger 1 mouse was placed inside the cylinder in the left compartment, with the cylinder on the right compartment empty. The test subject (*Mecp2* cKO mouse) was placed in the middle chamber for 10 min and its sniffing time towards stranger 1 and the empty chamber was recorded. At the end of the test, the test subject and stranger 1 were taken out and the chambers were cleaned. Ten minutes later, the test subject and stranger 1 were placed back to their original chambers, respectively. Meanwhile, a stranger 2 mouse was placed in the cylinder on the right compartment. The sniffing time of the test subject to stranger 1 and stranger 2 was recorded during the 10-min observation period, and it is regarded as the social novelty test. Social interaction behaviour was also measured in rats and the specifications for the three-chamber cage are 108 × 50 × 42 cm (*L* × *W* × *H*).

### Cued fear-conditioning learning

Fear-conditioning learning was preformed 7 days after the social interaction test. One day before conditioning, mice were placed in the conditioning chamber (46 × 30 × 46 cm, *L* × *W* × *H*) for 5 min for habituation. Twenty-four hours later, these animals were placed into the same chamber for fear-conditioning training. After 3 min of free exploration, they were trained with five tone-foot shock pairings. Each tone-shock pairing consisted of a 30-s tone (85 dB, 10 kHz), which is co-terminated with a foot shock (0.1 mA, 1 s). After shock presentation, a 60-s intertrial interval preceded the next tone and foot shock. Twenty-four hours later, these animals were placed into another chamber for the retention test. After 3 min of free exploration, a 30-s tone (85 dB, 10 kHz) was presented without foot shock. Five tones were given in the retention test with 60-s intervals. The freezing response of mice was calculated as the percentage of time spent freezing during the 30-s tone period. The parameters used for fear-conditioning learning were adopted from those of a previous study^64^.

### Immunohistochemistry

For immunohistochemical staining of PIAS1 and MeCP2 in the CA1 area of the rat brain, rats were anaesthetized with pentobarbital (100 mg kg^−1^, i.p.) and perfused with ice-cold PBS, followed by 4% paraformaldehyde. Brains were removed and post-fixed in 20% sucrose/4% paraformaldehyde solution for 20–48 h. Brains were then frozen, cut into 30-μm sections on a cryostat and mounted on gelatin-coated slides. Brain sections were rinsed with 1 × PBS for 10 min and permeabilized with pre-cold EtOH/CH_3_COOH (95%:5%) for 10 min, followed by 1 × PBS for 10 min for three times. The sections were pre-incubated in a blocking solution containing 3% normal goat serum, 3% bovine serum albumin (BSA) and 0.2% Triton X-100 in 1 × PBS for 2 h, followed by 1 × PBS for 10 min for three times. For visualization of endogenous PIAS1 and MeCP2 in hippocampal CA1 area, brain sections were incubated with rabbit anti-PIAS1 antibody (1:100, Catalogue No. 2474-1, Epitomics) and mouse anti-MeCP2 antibody (1:100, Catalogue No. H00004204-M01, Abnova, Taipei, Taiwan) at 4 °C overnight. The brain sections were then washed with 1 × PBS for 10 min for three times and then incubated with goat-anti-rabbit secondary antibody conjugated with FITC (1:500, Catalogue No. 111-095-003, Jackson Immunoresearch, West Grove, PA) and Cy3 donkey anti-mouse antibody (1:500, Catalogue No. GTX85338, Genetex) for 1 h. For immunofluorescence detection of the nucleus, tissue sections were added with 20 μl of the VECTASHIELD mounting medium with DAPI (1.5 μg ml^−1^; Vector Laboratories, Burlingame, CA). For examination of lentiviral vector transduction in BLA, brain sections were prepared for visualization of GFP (green) and RFP (red) fluorescence. Photomicrographs were taken using a Zeiss LSM510 confocal microscope.

### Primary hippocampal culture and immunocytochemistry

Embryonic primary hippocampal neurons were prepared from E18 of Sprague–Dawley rats. The hippocampus from the embryo was dissociated with 100 U ml^−1^ papain and plated onto 100 μg ml^−1^ poly-L-lysine-coated coverslips at a density of 3 × 10^5^ cells ml^−1^, with minimal essential medium containing 5% fetal bovine serum and 5% horse serum. Three hours later, the medium was replaced with neurobasal medium (Catalogue No. 21103049, Invitrogen) containing B27 supplements (Catalogue No. 17504044, Invitrogen), GlutaMAX supplement (Catalogue No. 35050061, Invitrogen), 100 units ml^−1^ Penicillin and 100 μg ml^−1^ Streptomycin (Catalogue No. 15140122, Invitrogen). Cultures were maintained at 37 °C in a humidified atmosphere at 5% CO_2_, and the neurobasal medium was replaced again 2 days later. For immunofluorescence visualization of PIAS1 and MeCP2 in dissociated neurons, cultured hippocampal neurons at DIV5 were fixed with 4% paraformaldehyde-4% sucrose (wt/vol) at room temperature for 10 min, the paraformaldehyde/sucrose mixture was then aspirated and 1 ml of 0.25% (vol/vol) Triton X-100 in PBS was added to each well at room temperature for 10 min. The coverslips were washed carefully with PBS and added with 1 ml of 5% (wt/vol) BSA in PBS at room temperature for 1 h. The primary antibodies, rabbit anti-PIAS1 antibody (1:300, Catalogue No. 2474-1, Epitomics) and mouse anti-MeCP2 antibody (1:300, Catalogue No. 61285, Active Motif) were diluted in 1% (wt/vol) BSA in PBS, added to the coverslip and incubated at 4 °C for overnight. The coverslips were then washed three times gently with PBS. After PBS washing, DyLight 488 goat-anti-rabbit antibody (1:1,000, Catalogue No. GTX76757, Genetex) and Cy3 donkey anti-mouse antibody (1:1,000, Catalogue No. GTX85338, Genetex) were diluted in 1% (wt/vol) BSA in PBS and added to the coverslips for 1 h. The coverslips were then washed three times with PBS and added with 20 μl VECTASHIELD mounting medium with DAPI (Vector Laboratories). Photomicrographs were taken using a Zeiss LSM510 confocal microscope.

### Statistics

Behavioural data were analysed with one-way or two-way analysis of variance (ANOVA) with repeated measure followed by *post hoc* Newman–Keuls multiple comparisons (represented by *q* value). Biochemical data were analysed with the Student's *t*-test or one-way ANOVA followed by Newman–Keuls comparisons. Electrophysiological data were analysed with two-way ANOVA with repeated measure followed by Newman–Keuls comparisons. Values of *P*<0.05 were considered statistically significant (*P<0.05, **P<0.01, ^#^*P*<0.001).

## Additional information

**How to cite this article:** Tai, D. J. C. *et al*. MeCP2 SUMOylation rescues *Mecp2*-mutant-induced behavioural deficits in a mouse model of Rett syndrome. *Nat. Commun.* 7:10552 doi: 10.1038/ncomms10552 (2016).

## Supplementary Material

Supplementary Information Supplementary Figures 1-11
