# Supplementary material for: MeCP2 SUMOylation rescues Mecp2-mutant-induced behavioural deficits in a mouse model of Rett syndrome
Source: Nat Commun. 2016 Feb 4;7:10552. doi: 10.1038/ncomms10552 (PMC4743023; doi:10.1038/ncomms10552)

Supplementary Figure S1

a

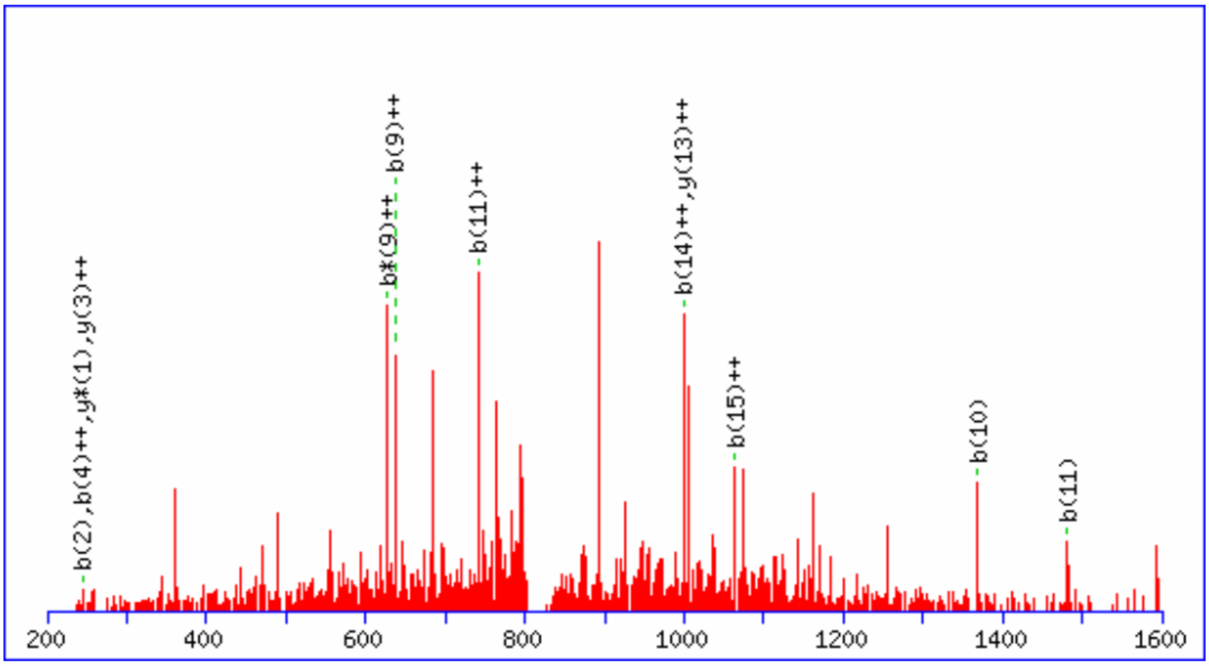

MeCP2 K22, K24 , K29, K32 candidate sumo sites

b

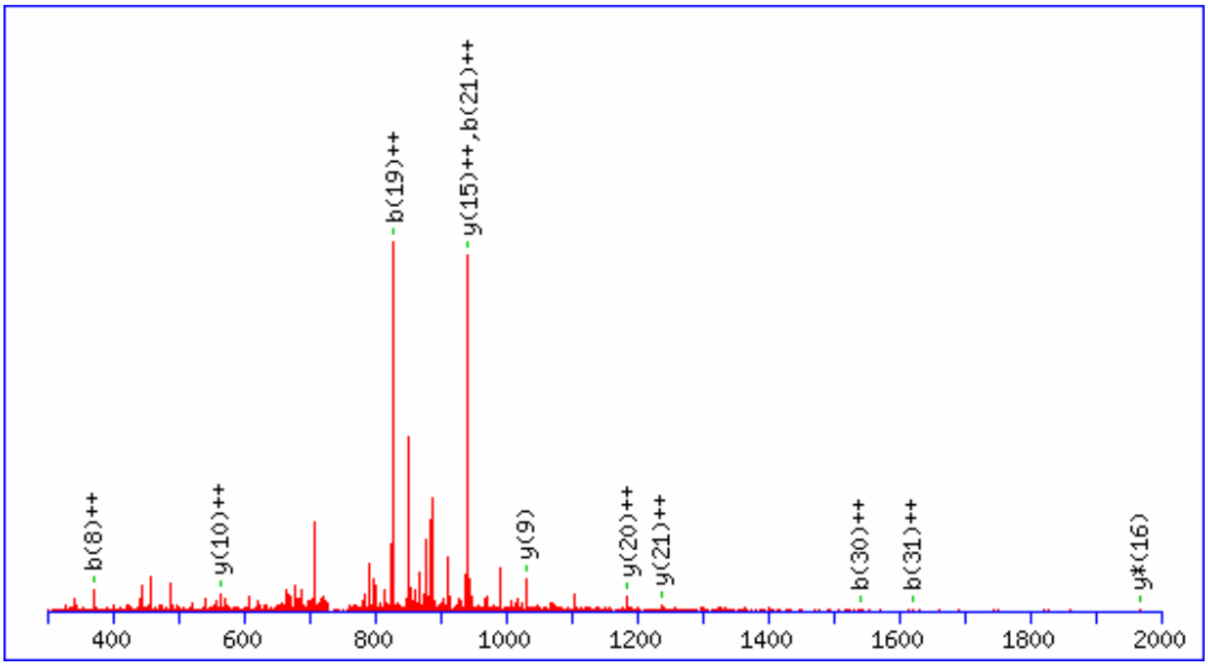

MeCP2 K249 candidate sumo site

Supplementary Figure S1 (continue)

c

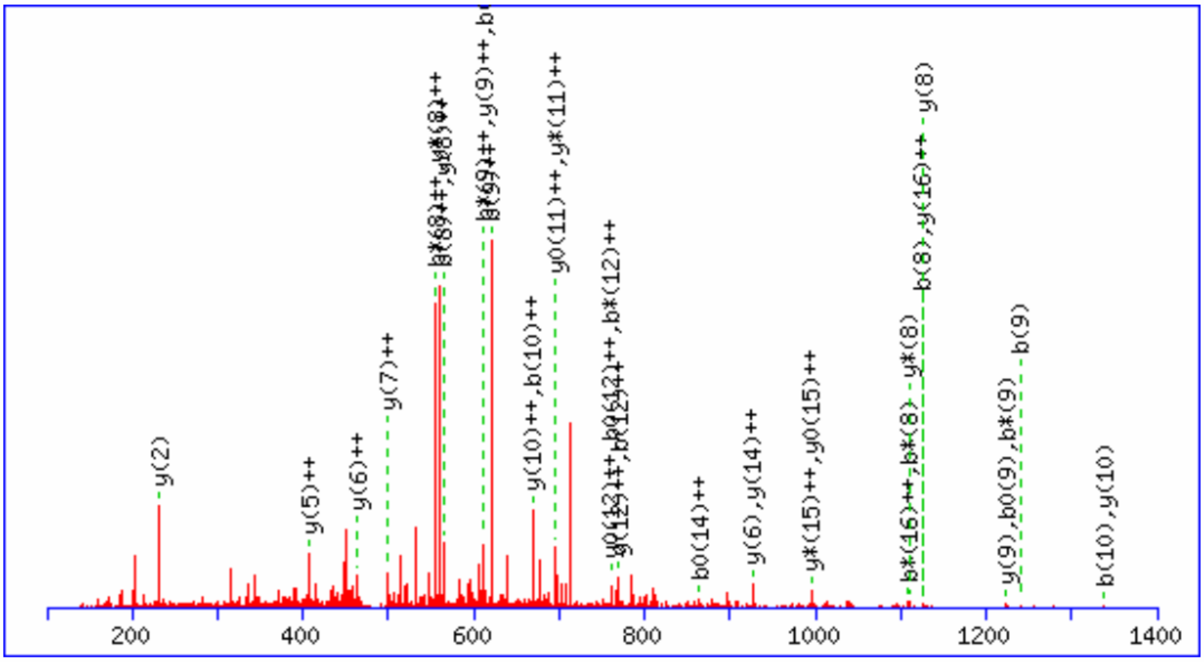

MeCP2 K254, K256 , K266, K267 candidate sumo sites

d

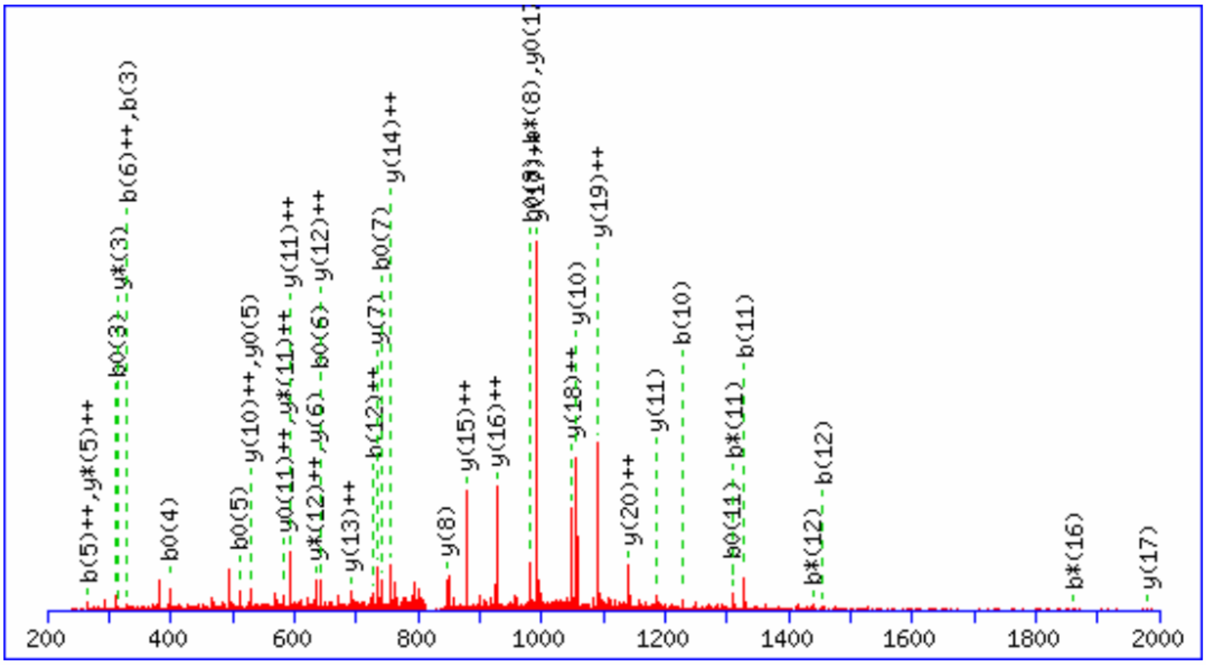

MeCP2 K317 candidate sumo site

Figure S1. LC-MS/MS prediction of potential SUMOylation sites on MeCP2. LC-MS/MS chromatography Prediction of potential SUMOylation sites on MeCP2 at (**a**) Lys-22, Lys-24, Lys-29, Lys-32 (**b**) Lys-249 (**c**) Lys-254, Lys-256, Lys-266, Lys-267 and (**d**) Lys-317. Results are from two independent experiments.

Supplementary Figure S2

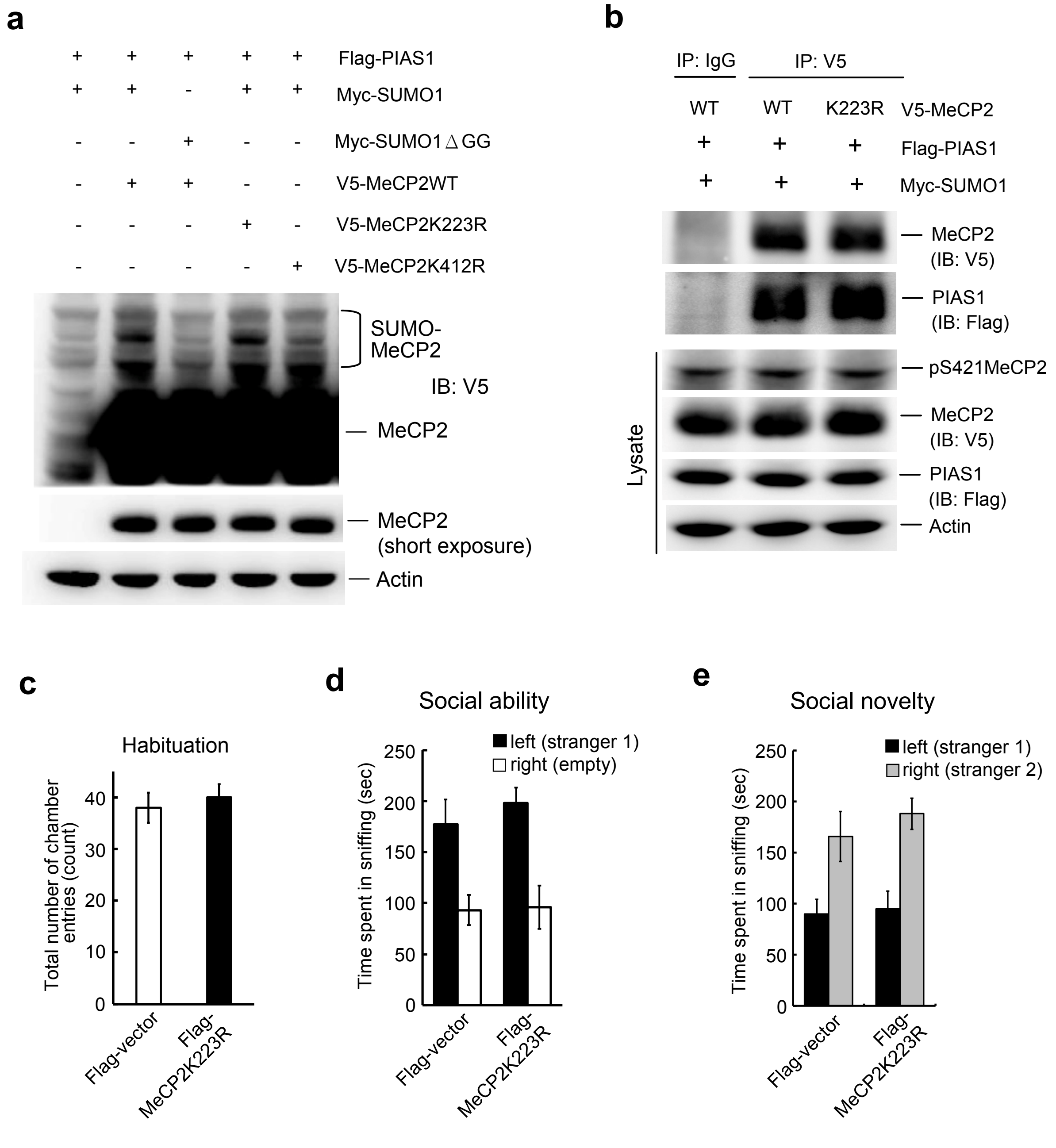

Figure S2. MeCP2 is not sumoylated by PIAS1 at Lys-223 and transfection of Flag-MeCP2K223R mutant plasmid does not affect social interaction behavior in rats. (**a**) Flag-PIAS1 plasmid was co-transfected with Myc-SUMO1 or Myc-SUMO1ΔGG plasmid, V5-MeCP2WT, V5-MeCP2K223R or V5-MeCP2K412R plasmid to HEK293T cells and MeCP2 SUMOylation was determined by western blot using anti-V5 antibody. V5-MeCP2K412R was used as a negative control. (**b**) Flag-PIAS1 plasmid was co-transfected with Myc-SUMO1 plasmid and V5-MeCP2WT plasmid (or V5-MeCP2K223R plasmid) to HEK293T cells and co-IP experiment was carried out 48 h later with immunoprecipitation using anti-V5 antibody and immunoblotting with anti-Flag antibody. The expression level of MeCP2 was also examined by western blot. The expression level of pS421MeCP2, MeCP2 and PIAS1 in cell lysate was also examined by western blot. Results are from two independent experiments. Flag-vector and Flag-MeCP2K223R plasmids were transfected to CA1 area in the rat brain. Social interaction behaviors including (**c**) motor activity (**d**) social ability and (**e**) social novelty were measured 48 h later (n=5 each group, $t_{1,8}$=0.52, $P > 0.05$ for total number of chamber entries; $t_{1,8}$=0.72, $P > 0.05$ for left stranger 1 of social ability measure; $t_{1,8}$=0.11, $P > 0.05$ for right empty of social ability measure and $t_{1,8}$=0.22, $P > 0.05$ for left stranger 1 of social novelty measure; $t_{1,8}$=0.78, $P > 0.05$ for right stranger 2 of social novelty measure, Student's *t*-test). Data are expressed as mean±SEM.

Supplementary Figure S3

a

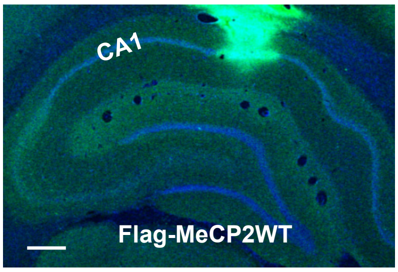

FITC-conjugated
secondary antibody

b

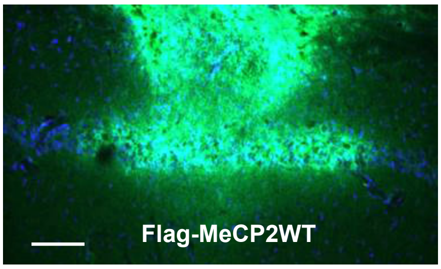

FITC-conjugated
secondary antibody

c

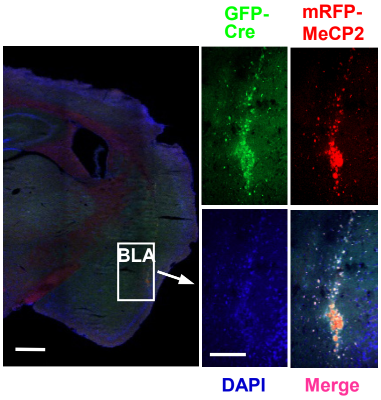

d

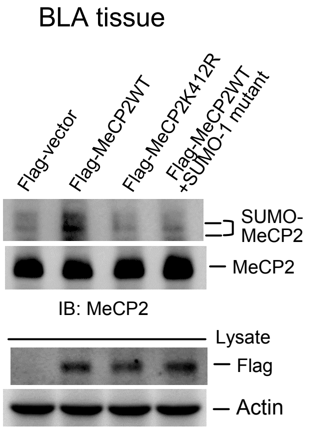

Figure S3. Immunohistochemistry showing plasmid transfection and expression in rat CA1 area and lentivector transduction and expression in the BLA. (**a**) Flag-MeCP2WT plasmid was transfected to CA1 area in the rat brain. The brain slice containing the CA1 area was subject to immunohistochemistry using anti-Flag antibody and FITC-conjugated secondary antibody. Cells that show green fluorescence were successfully transfected with the plasmid. DAPI (blue) was used for nucleus staining. Scale bar equals 400 μm. (**b**) The same tissue slice viewed at a higher magnification. Scale bar equals 100 μm. (**c**) Immunohistochemistry showing the location of recombinase *Cre* and mRFP-MeCP2WT transduction and expression, and their co-localization in BLA neurons. Scale bar equals 500 μm in the left panel and scale bar equals 100 μm in the right panels. (**d**) Flag-vector, Flag-MeCP2WT (with or without the addition of SUMO1 mutant protein) and Flag-MeCP2K412R were transfected to rat BLA area and *in vitro* SUMOylation assay was carried out to determine MeCP2 SUMOylation at Lys-412 in the amygdala.

Supplementary Figure S4

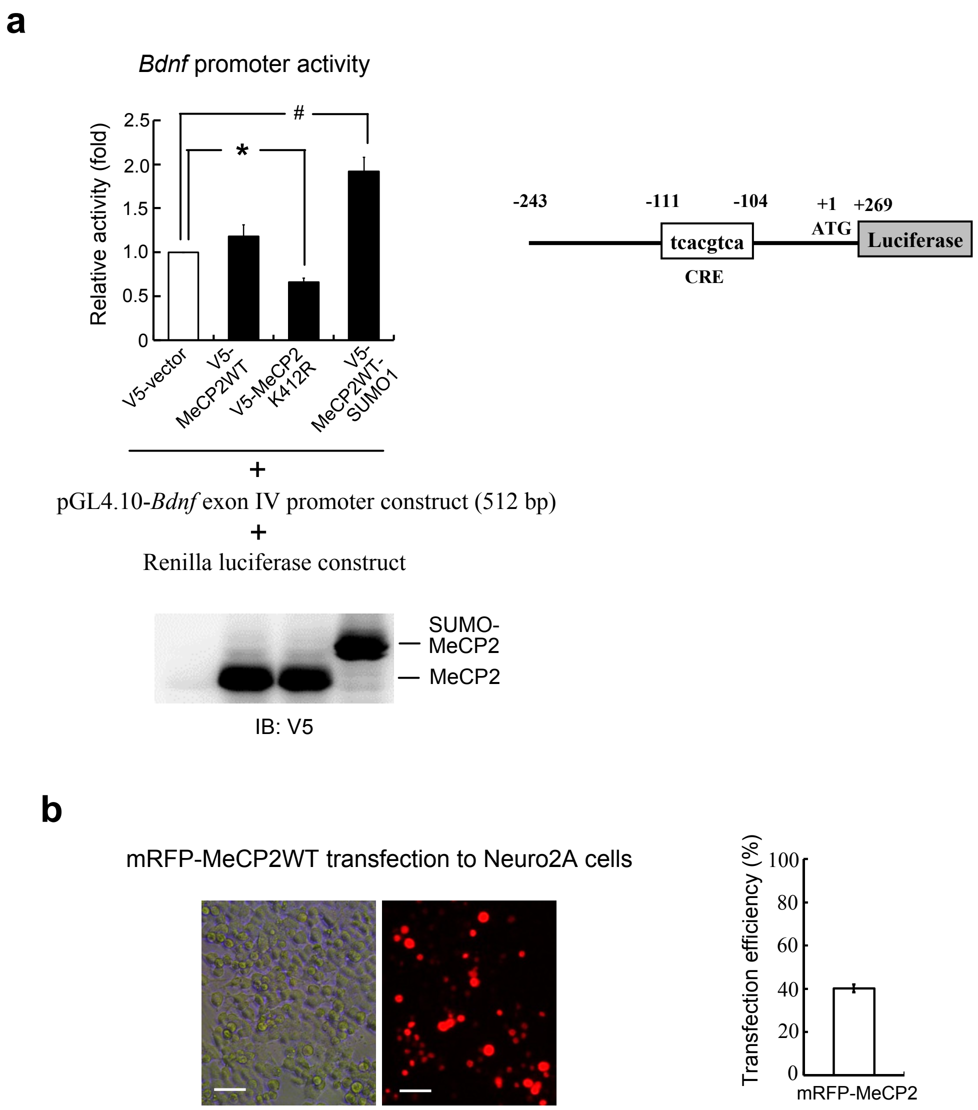

Figure S4. SUMOylation of MeCP2 enhances *Bdnf* promoter activity in Neuro2A cell. (**a**) V5-vector, V5-MeCP2WT, V5-MeCP2K412R or V5-MeCP2WT-SUMO1 fusion plasmid was co-transfected with the pGL4.10-*Bdnf* exon IV promoter construct and Renilla luciferase construct to Neuro2A cells and *Bdnf* promoter activity was determined by luciferase assay (Results are from four independent experiments, $F_{3,12}$=26.49, #P < 0.001; q=1.76, P > 0.05 comparing the V5-MeCP2WT group with V5-vector group; q=3.27, *P < 0.05 comparing the V5-MeCP2K412R group with V5-vector group; q=8.91, #P < 0.001 comparing the V5-MeCP2WT-SUMO1 group with V5-vector group, one-way ANOVA followed by the post-hoc Newman-Keul multiple comparisons). The *Bdnf* promoter construct containing the CRE element is shown in the right panel. Plasmid transfection and expression was confirmed by western blot using anti-V5 antibody. (**b**) For examination of plasmid transfection efficiency in Neuro2A cells, mRFP-MeCP2WT plasmid was transfected to Neuro2A cells; total number of cells (left panel) and the number of cells that showed red fluorescence (right panel) were counted 48 h later (upper panels). The ratio of these numbers was normalized and plotted in the lower panel. Scale bar equals 50 μm. Results are from three independent experiments. Data are expressed as mean±SEM.

Supplementary Figure S5

Up-regulated genes (RT-qPCR)

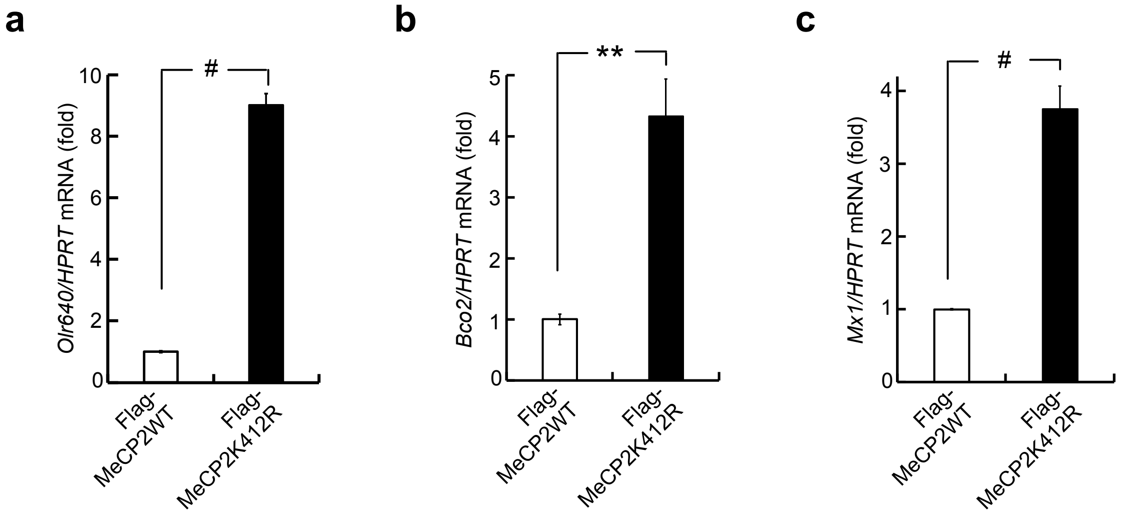

Down-regulated genes (RT-qPCR)

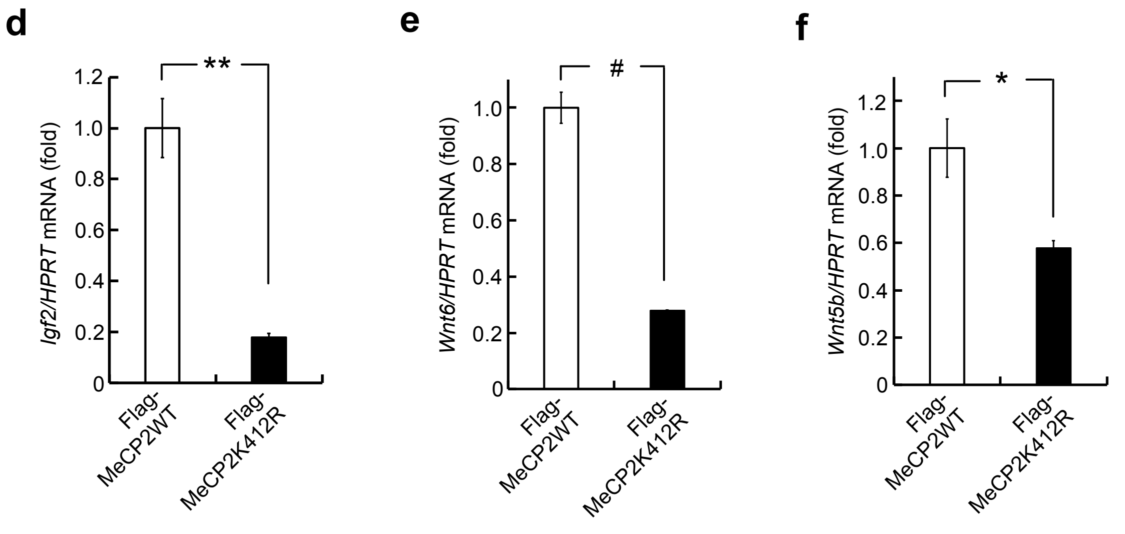

Figure S5. Reverse transcription-quantitative real-time PCR (RT-qPCR) of gene expression for selected genes identified from cDNA microarray analysis. Three genes that show up-regulation by Flag-MeCP2K412R transfection (a-c) and three genes that show down-regulation by Flag-MeCP2K412R transfection (d-f) from the microarray analysis were chosen for further RT-qPCR analysis. Flag-vector or Flag-MeCP2K412R plasmid was transfected to CA1 area in the rat brain (n=3 each group). Animals were sacrificed 48 h later and their CA1 tissue was subject to different sets of RT-qPCR analysis. (**a**) *Olr640* mRNA expression ($t_{1,4}$=21.69, #P < 0.001). (**b**) *Bco2* mRNA expression ($t_{1,4}$=5.4, **P < 0.01). (**c**) *Mx1* mRNA expression ($t_{1,4}$=8.59, #P = 0.001). (**d**) *Igf2* mRNA expression ($t_{1,4}$=7.0, **P < 0.01). (**e**) *Wnt6* mRNA expression ($t_{1,4}$=12.89, #P < 0.001). (**f**) *Wnt5b* mRNA expression ($t_{1,4}$=3.33, *P < 0.05). Data are expressed as mean±SEM.

Supplementary Figure S6

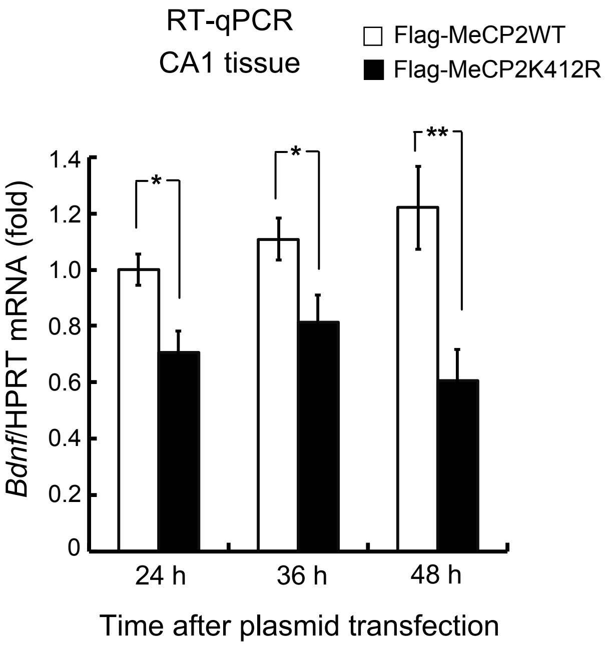

Figure S6. Time-course study of Flag-MeCP2K412R plasmid transfection on *Bdnf* mRNA expression. Flag-vector or Flag-MeCP2K412R plasmid was transfected to CA1 area in the rat brain. Animals were sacrificed at different time intervals (24 h, 36 h and 48 h) and their CA1 tissue was punched out for RT-qPCR determination of *Bdnf* mRNA expression. n=6 each group for each time interval examined ($t_{1,10}$=3.05, *P < 0.05 for 24 h interval; $t_{1,10}$=2.4, *P < 0.05 for 36 h interval; $t_{1,10}$=3.33, **P < 0.01 for 48 h interval). Data are expressed as mean±SEM.

Supplementary Figure S7

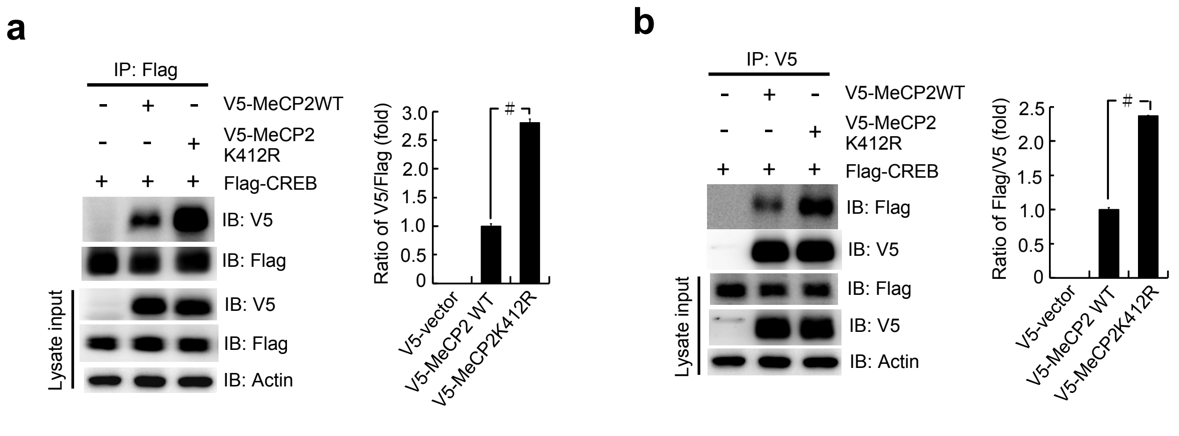

Figure S7. Blockade of MeCP2 SUMOylation increases the interaction of MeCP2 and CREB in HEK293T cells. (**a**) V5-MeCP2WT or V5-MeCP2K412R plasmid was co-transfected with the Flag-CREB plasmid to HEK293T cells and co-IP experiment was carried out with immunoprecipitation using anti-Flag antibody and immunoblotting using anti-V5 antibody. The expression level of CREB was examined by western blot. The quantified result is shown in the right panel (n=3 each group; $F_{2,6}$=1030.43, #P < 0.001; q=40.74, #P < 0.001 comparing the V5-MeCP2K412R group with V5-MeCP2WT group, one-way ANOVA followed by post-hoc Newman-Keul multiple comparisons). (**b**) The same plasmids were transfected to HEK293T cells and co-IP experiment was carried out as described in (a) except that cell lysates were immunoprecipitated with anti-V5 antibody and immunoblotted with anti-Flag antibody. The expression level of MeCP2 was examined by western blot. The quantified result is shown in the right panel (n=2 each group; $F_{2,3}$=4246.9, #P < 0.001; q=75.04, #P < 0.001 comparing the V5-MeCP2K412R group with V5-MeCP2WT group, one-way ANOVA followed by post-hoc Newman-Keul multiple comparisons).

Supplementary Figure S8

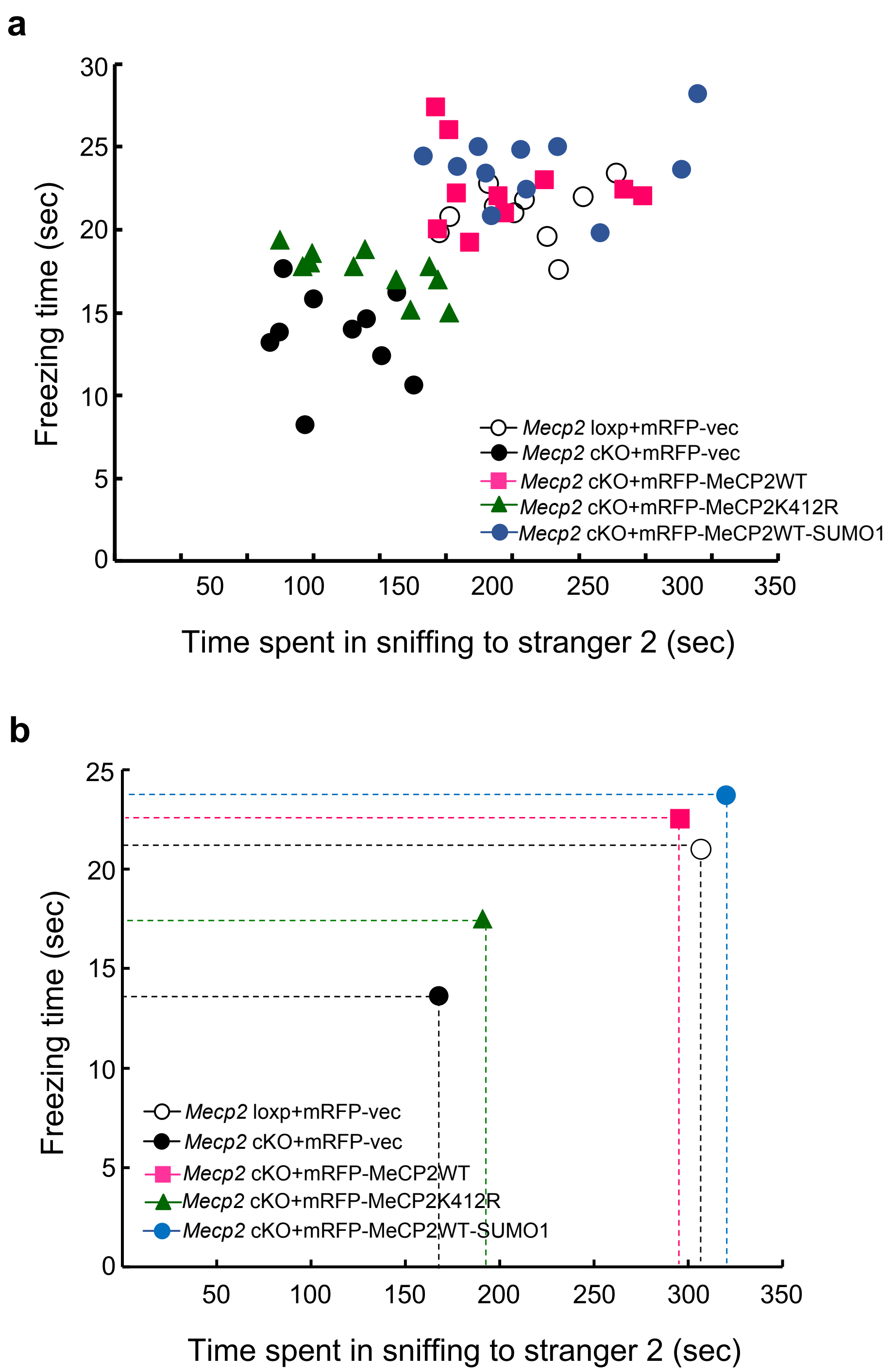

Figure S8. Correlation analyses between social novelty performance and cued fear memory performance in *Mecp2* cKO mice transducted with different MeCP2 lentiviral vectors. (**a**) Individual scores of time spent sniffing to stranger 2 during the social novelty test was plotted against individual scores of cued fear memory test for all the *Mecp2* cKO mice transducted with different MeCP2 lentiviral vectors and for *Mecp2* loxp control mice, respectively. (N=52 in total, r (correlation coefficient) = 0.64, $^{**}P < 0.01$). (**b**) Group means of time spent sniffing to stranger 2 during the social novelty test was plotted against group means of cued fear memory test for the same animals.

Supplementary Figure S9

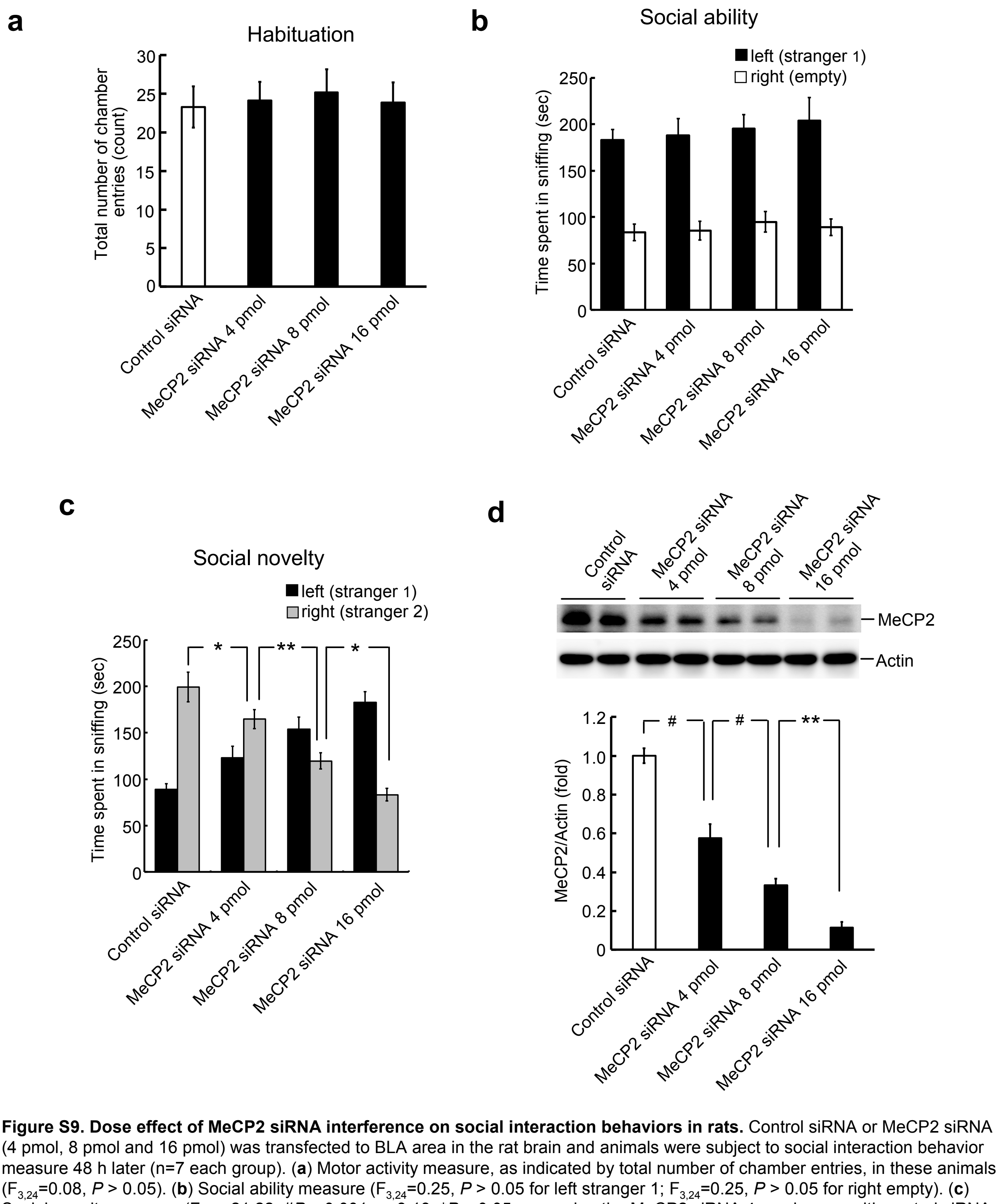

Figure S9. Dose effect of MeCP2 siRNA interference on social interaction behaviors in rats. Control siRNA or MeCP2 siRNA (4 pmol, 8 pmol and 16 pmol) was transfected to BLA area in the rat brain and animals were subject to social interaction behavior measure 48 h later (n=7 each group). (**a**) Motor activity measure, as indicated by total number of chamber entries, in these animals ($F_{3,24}$=0.08, P > 0.05). (**b**) Social ability measure ($F_{3,24}$=0.25, P > 0.05 for left stranger 1; $F_{3,24}$=0.25, P > 0.05 for right empty). (**c**) Social novelty measure ($F_{3,24}$=21.22, #P < 0.001; q=3.13, *P < 0.05 comparing the MeCP2 siRNA 4 pmol group with control siRNA group; q=4.09, **P <0.01 comparing the MeCP2 siRNA 8 pmol group with MeCP2 siRNA 4 pmol group; q=3.31, *P < 0.05 comparing the MeCP2 siRNA 16 pmol group with MeCP2 siRNA 8 pmol group). (**d**) Animals were sacrificed after the social novelty test. Their BLA tissue was punched out and subject to western blot analysis of MeCP2 expression ($F_{3,24}$=68.24, #P < 0.001; q=9.21, #P < 0.001 comparing MeCP2 siRNA 4 pmol group with control siRNA group; q=5.3, #P < 0.001 comparing MeCP2 siRNA 8 pmol group with MeCP2 siRNA 4 pmol group; q=4.75, **P < 0.01 comparing MeCP2 siRNA 16 pmol group with MeCP2 siRNA 8 pmol group, one-way ANOVA followed by the post-hoc Newman-Keul multiple comparisons). The quantified result is shown in the lower panel. Data are expressed as mean±SEM.

Supplementary Figure S10

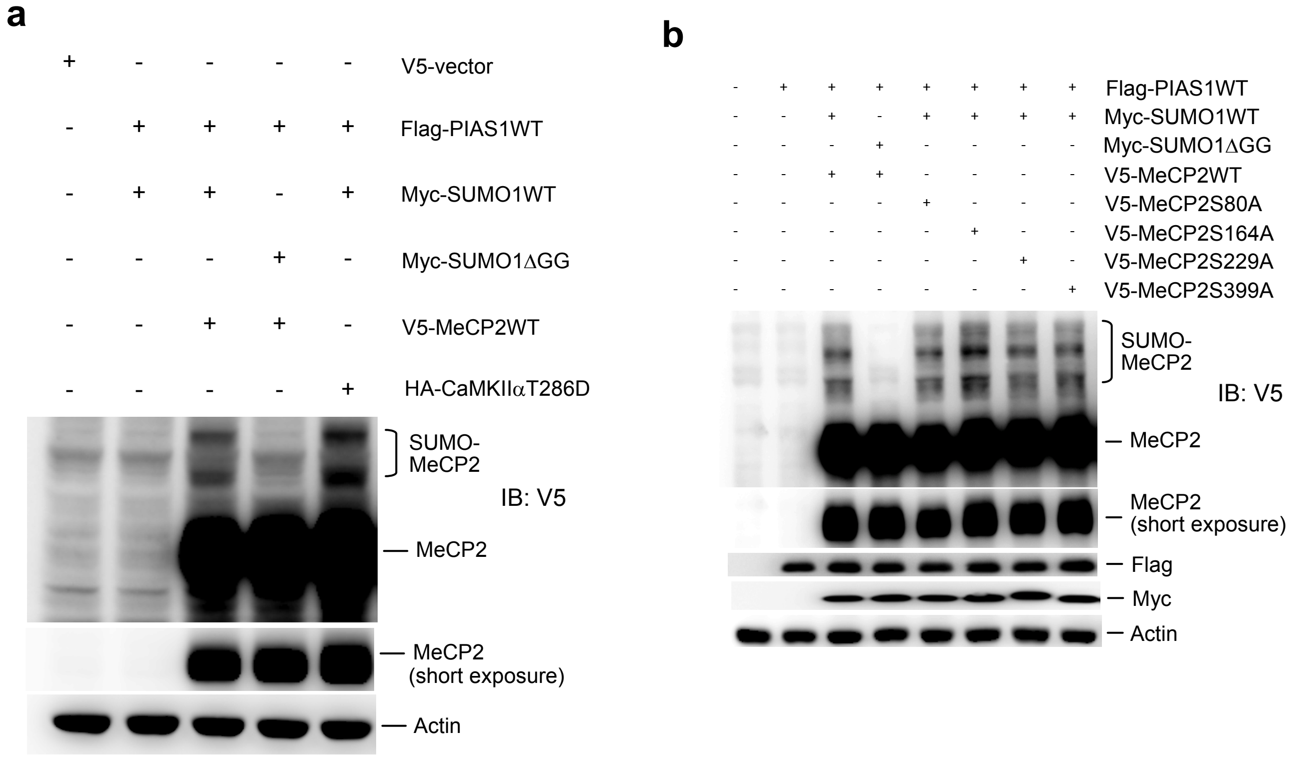

Figure S10. Transfection of constitutively active CaMKIIa enhances MeCP2 SUMOylation and mutation of MeCP2 at Ser-80, Ser-164, Ser-229 and Ser-399 does not affect MeCP2 SUMOylation. (**a**) V5-vector, Flag-PIAS1, Myc-SUMO1 or Myc-SUMO1ΔGG plasmids together with V5-MeCP2WT and HA-CaMKIIaT286D plasmids were transfected to HEK293T cells and MeCP2 SUMOylation was determined by western blot 48 h later using anti-V5 antibody. (**b**) Flag-PIAS1 and Myc-SUMO1 or Myc-SUMO1ΔGG plasmid together with V5-MeCP2WT or different V5-MeCP2 serine residue mutants were transfected to HEK293T cells and MeCP2 SUMOylation was determined by western blot 48 h later using anti-V5 antibody. Western blots against Flag and Myc were also carried out as loading controls. Results are from two independent experiments.

Supplementary Figure S11 (All full and un-cropped gels)

Fig. 1

b

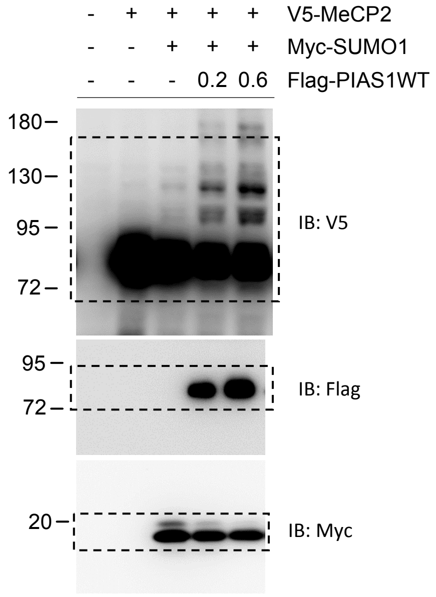

d

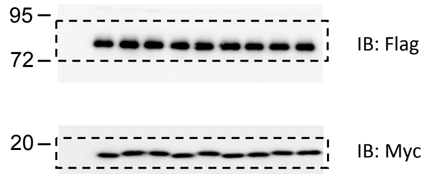

Fig. 2

a

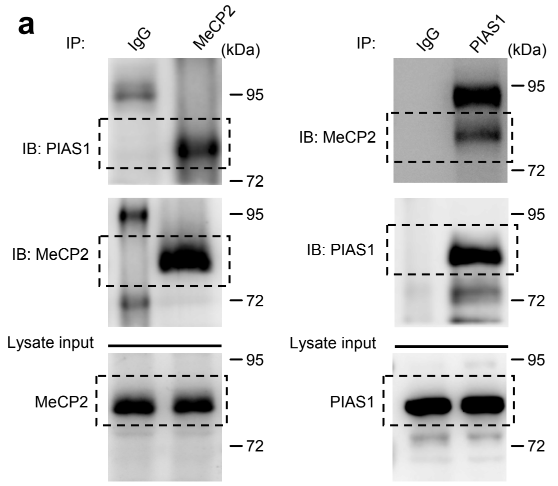

d

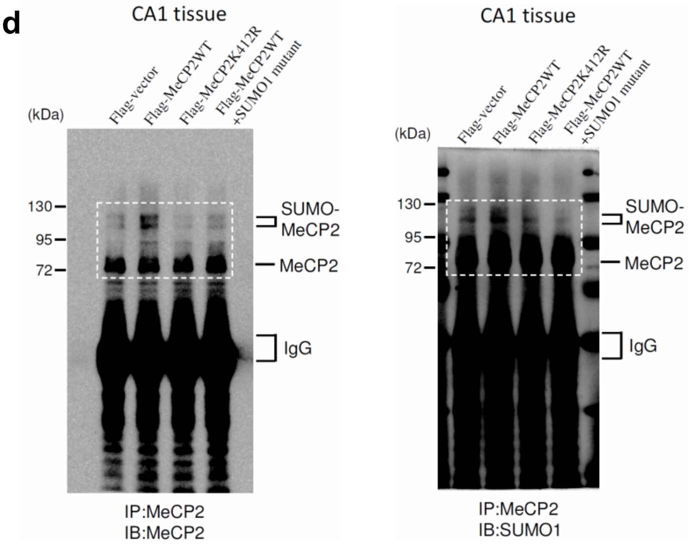

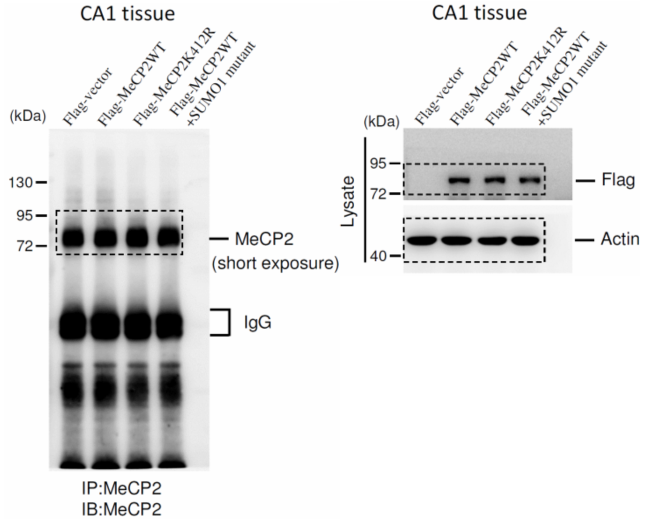

e

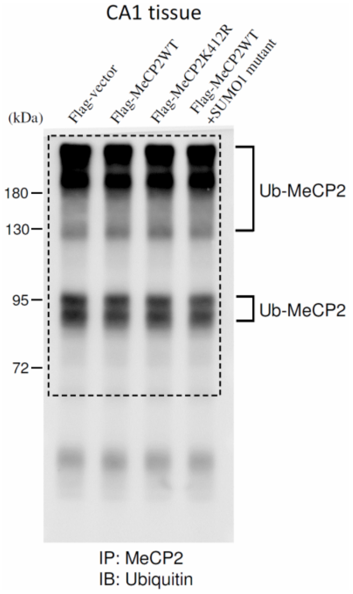

g

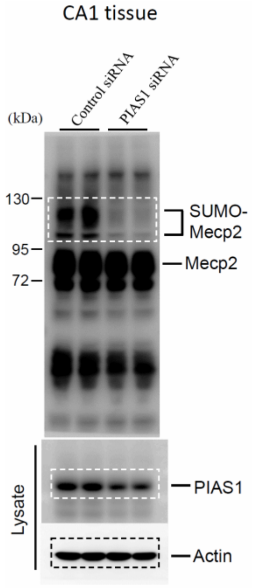

Supplementary Figure S11 (continue)

Fig. 3

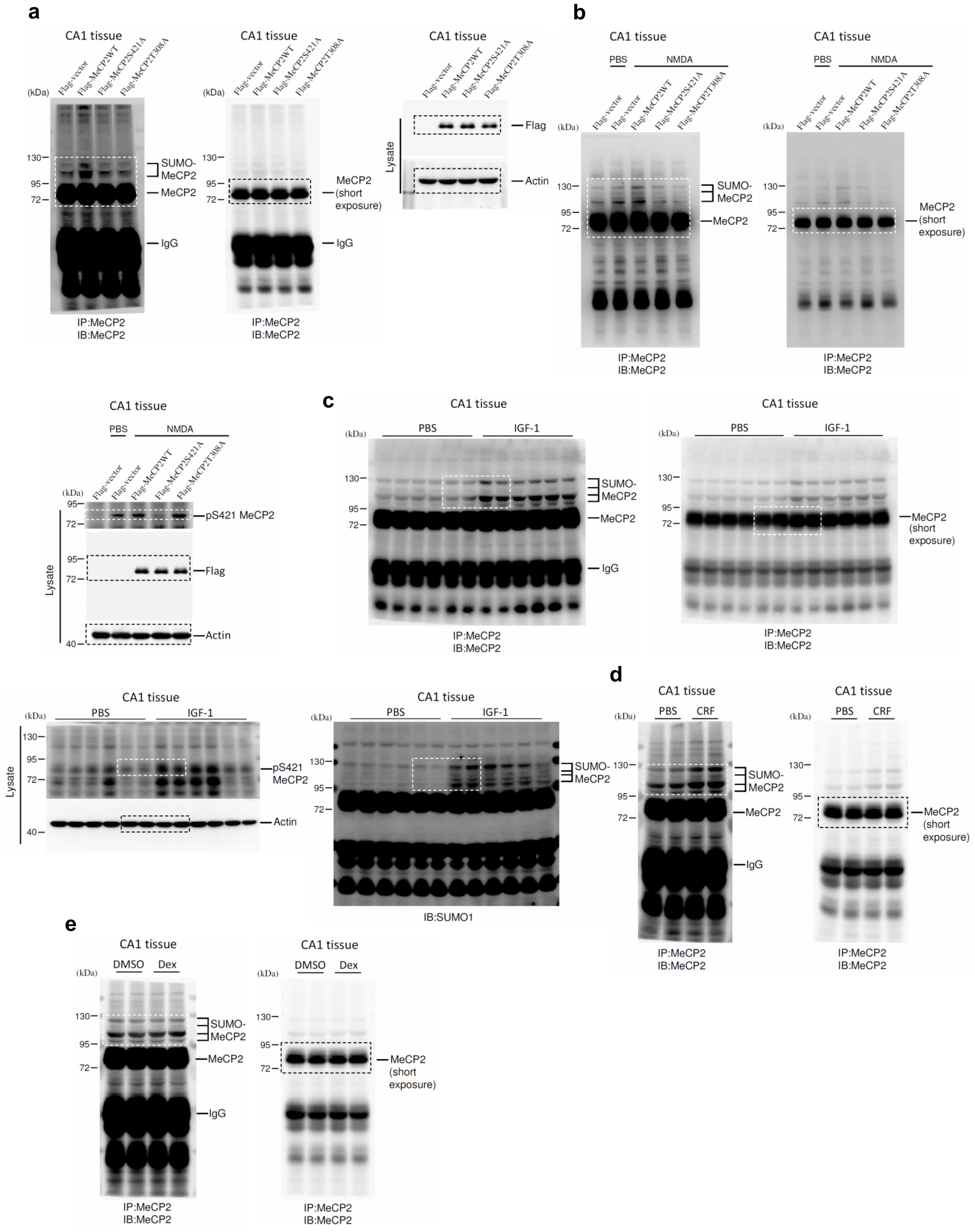

Supplementary Figure S11 (continue)

Fig. 4

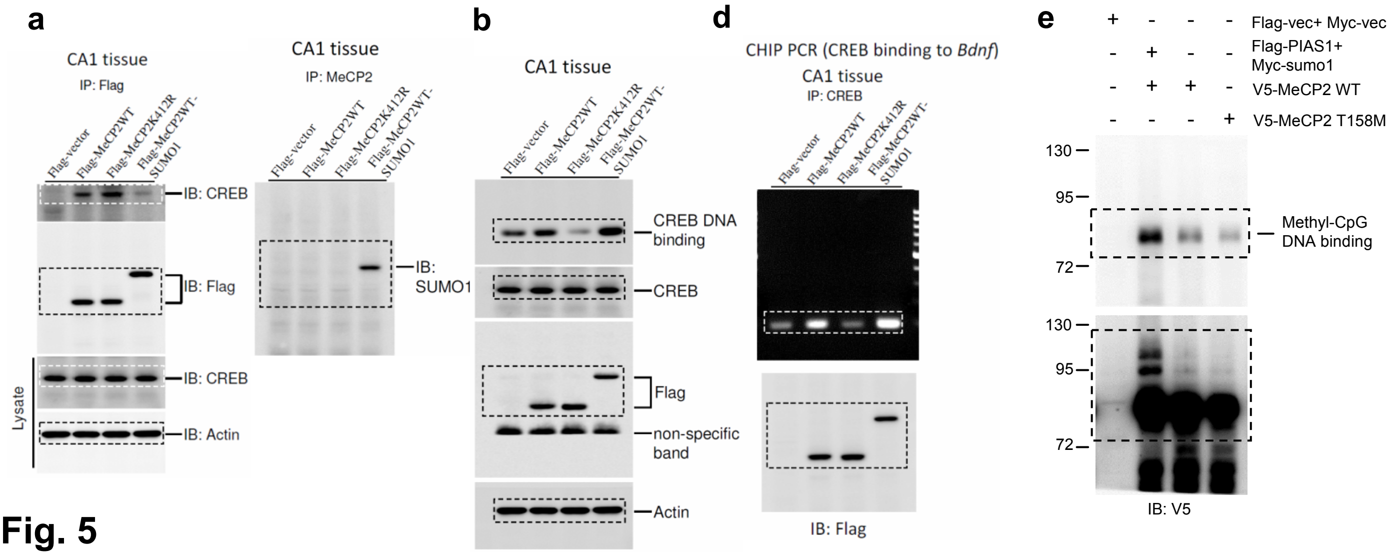

Fig. 5

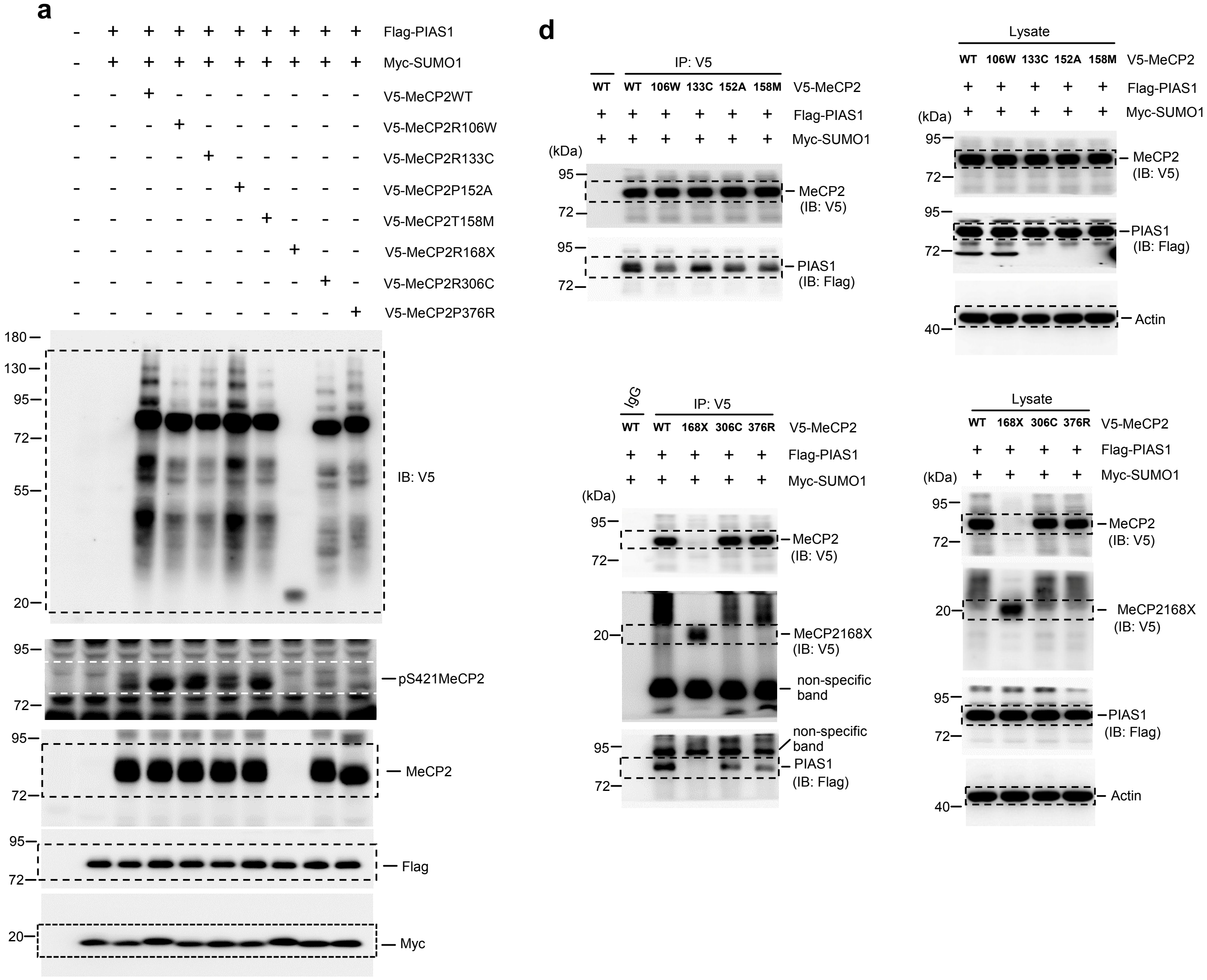

Supplementary Figure S11 (continue)

Fig. 6

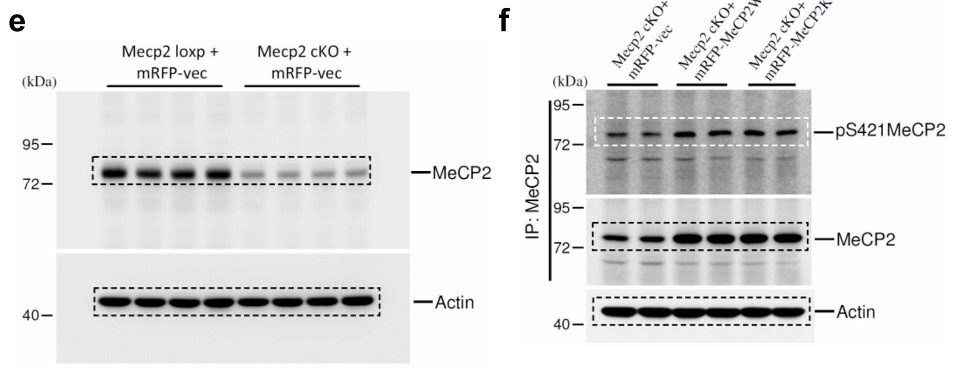

Supplement: Supplementary Information — Supplementary Figures 1-11 [file ncomms10552-s1.pdf]
